# Excellence in Communication and Emergency Leadership (ExCEL): Pediatric Critical Care Resource Utilization Workshop for Residents

**DOI:** 10.15766/mep_2374-8265.11268

**Published:** 2022-08-16

**Authors:** Robyn Wing, Hoi See Tsao, Marie Carillo, Laura Mercurio, Meghan Beucher, Linda Brown, Mariann Nocera Kelley

**Affiliations:** 1 Associate Professor, Departments of Emergency Medicine and Pediatrics, Division of Pediatric Emergency Medicine, Warren Alpert Medical School of Brown University and Rhode Island Hospital/Hasbro Children's Hospital; Director of Pediatric Simulation, Lifespan Medical Simulation Center; 2 Assistant Professor, Division of Pediatric Emergency Medicine, Department of Pediatrics, University of Texas Southwestern Medical Center; 3 Fellow, Department of Cardiology, Children's National Hospital; 4 Assistant Professor, Departments of Emergency Medicine and Pediatrics, Division of Pediatric Emergency Medicine, Warren Alpert Medical School of Brown University and Rhode Island Hospital/Hasbro Children's Hospital; 5 Assistant Professor, Departments of Emergency Medicine and Pediatrics, Division of Pediatric Emergency Medicine, Warren Alpert Medical School of Brown University and Rhode Island Hospital/Hasbro Children's Hospital; 6 Professor, Departments of Emergency Medicine and Pediatrics, Division of Pediatric Emergency Medicine, Warren Alpert Medical School of Brown University and Rhode Island Hospital/Hasbro Children's Hospital; Vice Chair of Pediatric Emergency Medicine, Brown Emergency Medicine; Director, Lifespan Medical Simulation Center; 7 Assistant Professor, Departments of Pediatrics and Emergency Medicine/Traumatology, Division of Pediatric Emergency Medicine, University of Connecticut School of Medicine and Connecticut Children's; Director of Simulation Education, University of Connecticut School of Medicine

**Keywords:** Defibrillator Use, Code Cart, Transport Bag, Critical Care Medicine, Clinical/Procedural Skills Training, Pediatric Emergency Medicine, Pediatrics

## Abstract

**Introduction:**

Efficiently locating critical equipment and prompt defibrillator usage are crucial steps when managing a critically ill patient or a code. However, resident experience in this area is limited. This workshop focused on the identification of critical care equipment in the pediatric code cart and transport bag along with timely, appropriate, and effective use of the defibrillator when needed.

**Methods:**

The workshop utilized a combination of traditional didactics and hands-on skills stations to instruct learners on the location of pediatric critical care equipment and the proper use of a defibrillator. It was designed for residents across all levels of training who care for pediatric patients (including pediatrics, medicine-pediatrics, triple board [pediatrics, psychiatry, and child psychiatry], family medicine, and emergency medicine residents) and can be adapted for different session durations and group sizes.

**Results:**

This workshop was conducted at two separate institutions, with a total of 95 resident participant encounters. Participants strongly agreed that the workshop was effective in teaching our learning objectives. Residents reported high levels of confidence in their ability to recognize and identify the location of critical care equipment in the code cart and transport bags and to appropriately use the defibrillator for both defibrillation and synchronized cardioversion.

**Discussion:**

This workshop provided residents with instruction and practice in locating and utilizing pediatric critical care equipment. The structure and timetable of this curriculum can be adapted to the needs of individual institutions’ programs and different numbers of workshop participants.

## Educational Objectives

By the end of this activity, learners will be able to:

1. Identify equipment contained in pediatric code carts.
2. Locate specific equipment in pediatric code carts for efficient use during resuscitations.
3. Identify equipment contained in the pediatric transport bag.
4. Locate specific equipment in the pediatric transport bag for efficient use during resuscitations.
5. Demonstrate appropriate steps for using the defibrillator for defibrillation and synchronized cardioversion.
6. Utilize the defibrillator appropriately in case scenarios.

## Introduction

Response time in the management of critically ill patients or pediatric arrests is of vital importance in determining patient survival and outcomes.^1^ Efficient resuscitation equipment access and usage are key components of decreasing resuscitation delays and improving patient care.^2^ Resuscitation equipment is usually found in dedicated carts and/or bags throughout the hospital and includes basic and advanced airway equipment, defibrillation equipment, and vascular access materials. Resuscitation situations are high-risk, high-stress, and low-frequency events for many residents.^3^ As a result, many residents do not develop skills, knowledge, or familiarity regarding access and use of resuscitation equipment in these situations during residency training, yet they are called on to be first responders during hospital emergencies.

Most critical resuscitation equipment is primarily located in code carts (also called resuscitation carts). Although there is no standard organization, most hospitals have code carts arranged by intervention type or patient weight to facilitate ease of access to equipment. Previous literature on code carts has shown that regular use, staff training and instruction, frequent retraining, and placement in every hospital department can improve patient outcomes during resuscitations.^4,5^ Although most residents caring for pediatric patients have received resuscitation training through the Pediatric Advanced Life Support (PALS) curriculum, there is no formalized training on resuscitation cart usage, despite residents’ role as first responders for critically ill pediatric patients. Evaluations from nursing students, medical students, and junior residents have expressed a desire for more practice with resuscitation cart components to improve skills and knowledge.^6^ Education and training with pediatric resuscitation carts greatly decrease the mean acquisition time for finding items in them, as well as increasing familiarity with them, although this knowledge fades over time.^2^ It is therefore important to have both effective initial trainings and periodic refresher courses on how to access components of pediatric resuscitation carts to avoid patient care delays and improve clinical outcomes.

Code carts are effective means of storing equipment for use when the patient is in a single location. However, critical resuscitation equipment is also needed when a patient is transported from one location to another within the same hospital. Intrahospital transport of critically ill patients is a common daily occurrence but represents a high-risk period for adverse events in up to 70% of intrahospital transports,^7–11^ of which 80% require therapeutic intervention^10^ and 34% are due to tools and technology.^12^ The majority of safety events are deemed preventable or possibly avoidable by staff training with transport equipment, double-checking of equipment before transport, and having experienced clinicians accompany the patient.^13^ Pediatric resident education surrounding the transport of pediatric patients varies widely and is not consistent between training programs.^14^ Additional exposure to the resources available for transport is vital to the care of critically ill patients.

Defibrillators are an additional piece of equipment needed for the care of critically ill patients. There are approximately 15,200 in-hospital pediatric cardiac arrests each year,^15^ 14%-43% of which are due to shockable presenting rhythms.^16^ Given the frequency of shockable rhythms, knowing how to effectively operate a defibrillator is a vital skill for leading a resuscitation. Residents who care for pediatric patients are trained in PALS, which includes very brief training in defibrillator use. However, knowledge obtained during PALS training declines over the first 12 months of residency.^17^ Furthermore, pediatric cardiac arrests are rare compared to adult in-hospital cardiac arrests^16^ and thus provide fewer opportunities to gain experience during residency training. Hunt and colleagues surveyed pediatric residents and reported that only 16% had ever discharged a defibrillator on a patient, while one-third had never used a defibrillator on a patient, even during practice scenarios.^18^ Meanwhile, it has been shown that simulation sessions increase the confidence level and comfort of pediatric residents in code scenarios.^19^ Furthermore, Andreatta and colleagues showed a correlation between initiation of a resident mock code curriculum and improvement in pediatric patient survival outcomes following cardiopulmonary arrest.^20^

An understanding of the resources available during the care of a critically ill patient is of the utmost importance to the care of the patient. Most important among these resources are those resources needed for immediate intervention, including equipment contained in the code cart or transport bag and the defibrillator. There are no published curricula designed specifically to instruct residents on the use of pediatric critical care equipment. There is one *MedEdPORTAL* publication that focuses on orienting pediatric residents to emergency care equipment including locating the code cart.^21^ However, this educational exercise is not case based and does not include the use of pediatric critical care equipment. There is a particular gap in training on the use of the code cart and equipment needed for intrahospital pediatric transports. Furthermore, while there are published cardiac arrest simulation modules for pediatric residents that incorporate defibrillator use in mock code scenarios,^22,23^ there are no dedicated workshops published that allow pediatric residents to repetitively practice defibrillator operation.

We therefore developed this pediatric critical care resources workshop to include a pediatric code cart skills session, pediatric transport bag skill session, and defibrillator use skills session to teach trainee recognition of the equipment contained within the code cart and transport bag and improve comfort with defibrillator use.

This workshop is part of a series from our larger curriculum called Excellence in Communication and Emergency Leadership, or ExCEL.^24,25^ The ExCEL curriculum was developed with the goal of replacing standard resident morning-report didactics once a month with simulation-based training and core resuscitation skills sessions. The ExCEL curriculum aims to augment the clinical skills obtained through training, while improving resident confidence, sharpening and maintaining critical technical and leadership skills, and improving communication proficiency. Monthly small-group workshops leverage the characteristics and advantages of social learning theory, active learning, and adult learning theory by coupling case-based learning with active commitment and engagement exercises.^19,26^ Our pediatric critical care resource utilization skills sessions can be used independently or in conjunction with other sessions from the ExCEL curriculum.

## Methods

There were three skills sessions in this longitudinal workshop series: the code cart skills session, the transport bag skills session, and the defibrillator use skills session.

### Target Audience

The target audience for our curriculum included residents in pediatrics, medicine-pediatrics, triple board (pediatrics, psychiatry, and child psychiatry), emergency medicine, and family medicine programs. Other learners who often attended the educational sessions included rotating third- and fourth-year medical students, but they were not our target audience. We ran this workshop at two different institutions, adapting the skills sessions to the needs and structure of the morning report at each. Prior participant knowledge of pediatric rhythm recognition via PALS training was beneficial, but not necessary, for participants.

### Instructor/Facilitator

The instructors for this workshop were pediatric emergency medicine faculty members, pediatric emergency medicine fellows, pediatric chief residents, or pediatric critical care transport paramedics or nurses who possessed knowledge of PALS as well as institution-specific code carts, transport bags, and defibrillators. When possible, multiple instructors were present to allow for a dedicated instructor to troubleshoot any technical difficulties that participants might experience and to facilitate multiple simultaneous hands-on sessions.

### Setting

The setting differed depending on available space and included either a large conference room or a patient care room. The only requirement was a space large enough for the equipment and number of scheduled learners, while adhering to COVID-19 precautions in accordance with institutional requirements.

### Timetable

Each skills session was 30 minutes long and had five to 10 participants. Participant numbers were limited due to the hands-on nature of the workshops and institution-specific COVID-19 restrictions. These sessions took place during typical morning-report hours for residents. For more details, refer to Table 1.

**Table 1.**
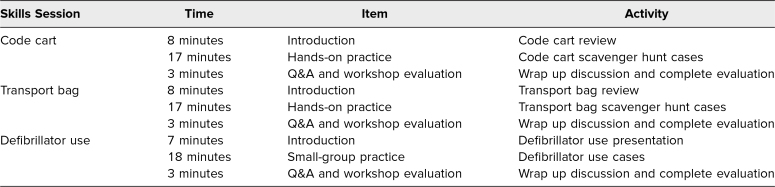
Suggested Timetable for Pediatric Critical Care Resource Utilization Workshop

### Equipment

In advance of the workshop, instructors identified and secured the necessary equipment for the skills sessions:

• Code cart skills session
  ○ Pediatric code cart with equipment (institution specific)
  ○ Defibrillator (e.g., Zoll)
  ○ Low- or medium-fidelity manikin (e.g., Laerdal Sim Junior)
• Transport bag skills session
  ○ Pediatric transport bag with equipment (institution specific)
  ○ Low- or medium-fidelity manikin (e.g., Laerdal Sim Junior)
• Defibrillator use skills session
  ○ Medium-fidelity manikin (e.g., Laerdal Sim Junior) allowing for assessment of chest rise with bag-valve-mask, CPR, and cardiac rhythm generation
  ○ Defibrillator(s) (e.g., Zoll): number of defibrillators needed depended on number of small groups
  ○ Rhythm generator (e.g., Nasco Life/form)

On the day of the skills sessions, instructors arrived early to ensure that the code cart and transport bag had all equipment present and for setup and testing of the defibrillator and rhythm generator. The instructor for the defibrillator use presentation (Appendix A) ensured that the audiovisual equipment was working properly. The rooms were set up to give residents space to gather around the medical equipment for optimal viewing. A manikin on a stretcher or table was available for case scenarios.

### Content

#### Code cart skills station

This session began with an instructor providing a tour of the pediatric code cart, pointing out where critical care items were located. Next, residents were divided into small groups to go on a scavenger hunt for necessary items to care for a patient as described in the clinical case vignettes (Appendix B).

#### Transport bag skills station

This session began with an instructor providing a tour of the pediatric transport bag, pointing out where critical care items were located. Next, residents were divided into small groups of three to five learners to go on a scavenger hunt for necessary items to care for a patient as described in the clinical case vignettes (Appendix C). Only one of the participating institutions had a transport bag, and so, this skills station was conducted at only that institution.

#### Defibrillator use skills station

This session began with an introductory presentation given via PowerPoint (Appendix A) to the group. The presentation (Appendix A) was a brief tutorial and discussion about the operation of the defibrillator. It included a demonstration of how to attach pads to the defibrillator and the patient (manikin), how to change to different modes of the defibrillator, an orientation to the buttons on the machine, and step-by-step instructions on how to defibrillate or cardiovert a patient. We provided an outline of these steps (Appendix D) in case the instructor wanted to use a hands-on demonstration, rather than PowerPoint, with a smaller group of participants. After this overview, the instructor used a rhythm generator to display various cardiac rhythms on the defibrillator. Participants were asked individually to use the defibrillator for different patient scenarios (Appendix E). Appendix F is a PowerPoint that includes the cases and rhythm strips should an institution not have a rhythm generator available to display particular rhythms on the defibrillator directly.

### ExCEL Curriculum

As previously mentioned, this workshop was part of a series from our larger ExCEL curriculum.^24,25^ As an unordered series, these skills sessions could be given at any time in the curriculum. The sessions were usually planned 6 months in advance but could be adjusted accordingly if facilitators identified a clinical concern on a relevant topic that could benefit from an educational intervention. The workshop was typically run annually.

### Evaluation

Residents were asked to complete the ExCEL critical care resources workshop surveys (Appendix G) as an evaluation of each skills station and to provide feedback on our goals and objectives as perceived by the learners. This form also solicited feedback about participants’ suggestions for improvement. The survey evaluation questions were developed by the facilitators based on the learning objectives for the skills station and used the format of the evaluations for previous ExCEL workshops.^24,25^ For ease of use and to maximize response rates, we converted the survey into an electronic form that was easily accessible with a QR code.

## Results

This workshop was conducted at two separate institutions as part of the ExCEL curriculum. A total of 95 residents participated across all three skills stations at both institutions, 64 from institution A and 31 from institution B. Each iteration of the skills stations included four to 12 resident participants with one to two facilitators. All 95 participants in the skills stations completed the postsession evaluation survey.

Of the 95 total participants, 36 (38%) were interns (PGY 1), 58 (61%) were residents (PGY 2 and above), and one (1%) declined to answer. Seventy-four (78%) were from pediatric residencies, 10 (11%) were from emergency medicine residencies, 10 (11%) were from family medicine residencies, and one (1%) declined to answer. Twenty-seven (28%) had completed a PALS course within the preceding 6 months, 32 (34%) had completed PALS 6 months to 1 year prior, and 33 (35%) had completed PALS 1-2 years prior.

We received very positive feedback about learner satisfaction and self-efficacy to perform the tasks in the educational objectives. Residents reported that each skills station within the workshop was effective at meeting the learning objectives and was relevant to their work (Table 2). Residents also reported confidence in their ability to recognize the equipment needed to care for a critically ill patient, identify and locate equipment in the code cart or transport bag, identify PALS rhythms, differentiate between shockable and nonshockable rhythms, and appropriately use the defibrillator for defibrillation and synchronized cardioversion (Table 3). The data were similar between the two institutions and therefore are presented only in the aggregate.

**Table 2.**
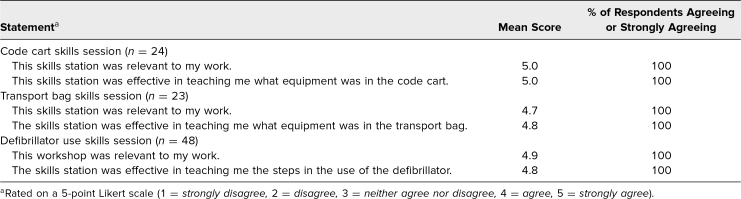
Participant Feedback on Relevance to Training and Attaining Learning Objectives

**Table 3.**
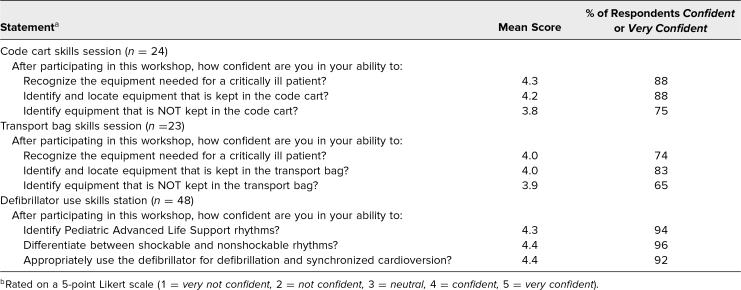
Participant Feedback on Self-Confidence in Relation to Learning Objectives

The residents reported that the scenarios and repetition were helpful in prompting them to find the equipment in the code cart and transport bags and that practice with real-time feedback was the most helpful. They also felt that the ability to have hands-on practice with the defibrillator and the reinforcement of rhythm recognition skills through repetition were most helpful.

Suggestions for future iterations of the skills stations included allowing more time for the defibrillator and rhythm recognition skills station and providing additional scenarios focused on medications that were included and not included in the code cart and transport bag.

Representative participant quotes included the following:

• “Such a helpful session. I now feel like I would know what to grab in the first five minutes and how to find equipment for others down the line. Really helpful to run through the scenario and discuss prioritization during a code.”
• “The scenarios were very helpful because we got to gather the equipment which reinforced what was in the bag.”
• “It was helpful opening the transport bag for the first time.”
• “It was helpful to review the different rhythms and PALS algorithms.”
• “Interactive and engaging.”

## Discussion

Providing medical care for critically ill patients requires efficient use of critical care resources. These resources include basic and advanced airway equipment, defibrillator equipment, and vascular access materials, which are kept in specific locations inside code carts and transport bags. Due to the infrequent nature of pediatric codes, residents caring for hospitalized children have little experience finding and using this equipment.^6,13,18^ PALS training and recertification are essential. However, skills decline when residents are not given the opportunity to use them, highlighting the need for reinforcement between PALS certification cycles.^17^

This workshop, consisting of three individual 30-minute skills stations, provides an effective means of training residents to locate and use necessary equipment to care for critically ill patients. After our workshop, the majority of residents felt confident in their ability to recall the equipment needed to care for a critically ill pediatric patient, identify and locate this equipment in either the code cart or transport bag, identify equipment not located in either the code cart or transport bag, and appropriately use the defibrillator for defibrillation and synchronized cardioversion. Qualitative feedback directly after each session was overall positive. However, instances even more illustrative of the usefulness of the sessions came weeks and months later when residents and faculty reported real-life examples of residents’ ability to access these critical materials quickly and more easily when needed for patient care.

With each iteration of this workshop, we learned that the equipment needs were determined by the number of participants and available resources. For example, when a rhythm generator for the defibrillator use skills station was not available, a medium- or high-fidelity manikin could be used to generate a rhythm on the defibrillator. Alternatively, if neither of these was available, rhythms could also be displayed via PowerPoint (Appendix F), and residents could still have hands-on practice with a defibrillator. Our two institutions utilized different defibrillator models. Therefore, we recognized the importance of including various defibrillator models in our presentation (Appendix A). Additionally, the groups were kept small in part due to COVID-19 restrictions and in part due to the importance of limiting group size to allow individuals hands-on practice. We found small groups of five to 10 to be the optimal number of participants.

A key limitation of the workshop involved catering to the diversity of participants’ prior critical care experiences based on their residency program and level of training. Residents from different programs (pediatrics, medicine-pediatrics, triple board, family medicine, and emergency medicine) have different experiences and familiarity with hospital resources, pediatric cardiac arrest management, dysrhythmias, and defibrillator use. As a result, this workshop may be of varying educational utility for residents based on their training specialty. While our project was not powered to assess these differences, overall feedback from learners was overwhelmingly positive. Instructors can account for these differences by soliciting feedback on knowledge and skill gaps prior to and during the session and tailoring to specific educational needs.

A limitation specific to the code cart skills station involved institutional variation in code cart organization. This precluded the use of standardized materials when orienting learners to the code cart and required a full code cart to be available for the skills station. When an actual code cart could not be obtained due to patient care needs or hospital restrictions, an identical, education-only code cart was substituted and still provided hands-on experience for the residents. Educational evaluation of the transport bag skills session was limited due to only one institution utilizing a preestablished transport bag. As above, if an actual transport bag was not available, an education-only transport bag was utilized.

A significant limitation in the defibrillator use skills station was time constraints. As a result, the station focused on defibrillation and synchronized cardioversion rather than the use of the defibrillator for transthoracic cardiac pacing, an extremely rare event in pediatrics. If time allowed, an additional 10 minutes for the session would have permitted more exploration into the rhythms presented. Additional time may be needed to include cardiac pacing education. Finally, this workshop is not designed to completely reeducate on the PALS concepts of cardiac arrest. However, it can serve to reinforce the formalized training provided by PALS.

This workshop has been designed as part of the larger ExCEL curriculum and can be implemented as a component of a larger morning-report curriculum or can stand on its own as an independent educational tool. The workshop augments PALS training by providing a guided opportunity for trainees to refresh their PALS skills between recertification sessions as well as a systematic framework to approach the equipment, communication, and leadership required to care for a pediatric patient in cardiac arrest. Preliminary findings show that the training provided in the workshop improves resident confidence in utilizing pediatric critical care resources. Additional quantitative and qualitative data from both mock and actual pediatric resuscitations are currently being reviewed to further evaluate and optimize this curriculum. Based on resident feedback, the next iteration of this workshop will allow for more hands-on time with the defibrillator as well as additional cases for enhanced knowledge and skill application.

## Appendices


Defibrillator Use Presentation.pptxCode Cart Skills Station.docxTransport Bag Skills Station.docxIntroduction to Defibrillator.docxDefibrillator Use Skills Station Cases.docxDefibrillator Use Skills Session Rhythm Strips.pptxExCEL Critical Care Workshop Surveys.docx
*All appendices are peer reviewed as integral parts of the Original Publication.*
