# Supplementary material for: Excellence in Communication and Emergency Leadership (ExCEL): Pediatric Critical Care Resource Utilization Workshop for Residents
Source: MedEdPORTAL. 2022 Aug 16;18:11268. doi: 10.15766/mep_2374-8265.11268 (PMC9378690; doi:10.15766/mep_2374-8265.11268)
Supplement: Supplementary file 1 — Defibrillator Use Presentation.pptxCode Cart Skills Station.docxTransport Bag Skills Station.docxIntroduction to Defibrillator.docxDefibrillator Use Skills Station Cases.docxDefibrillator Use Skills Session Rhythm Strips.pptxExCEL Critical Care Workshop Surveys.docx [file mep_2374-8265.11268-s001.zip › A. Defibrillator Use Presentation.pptx]

## Slide 1
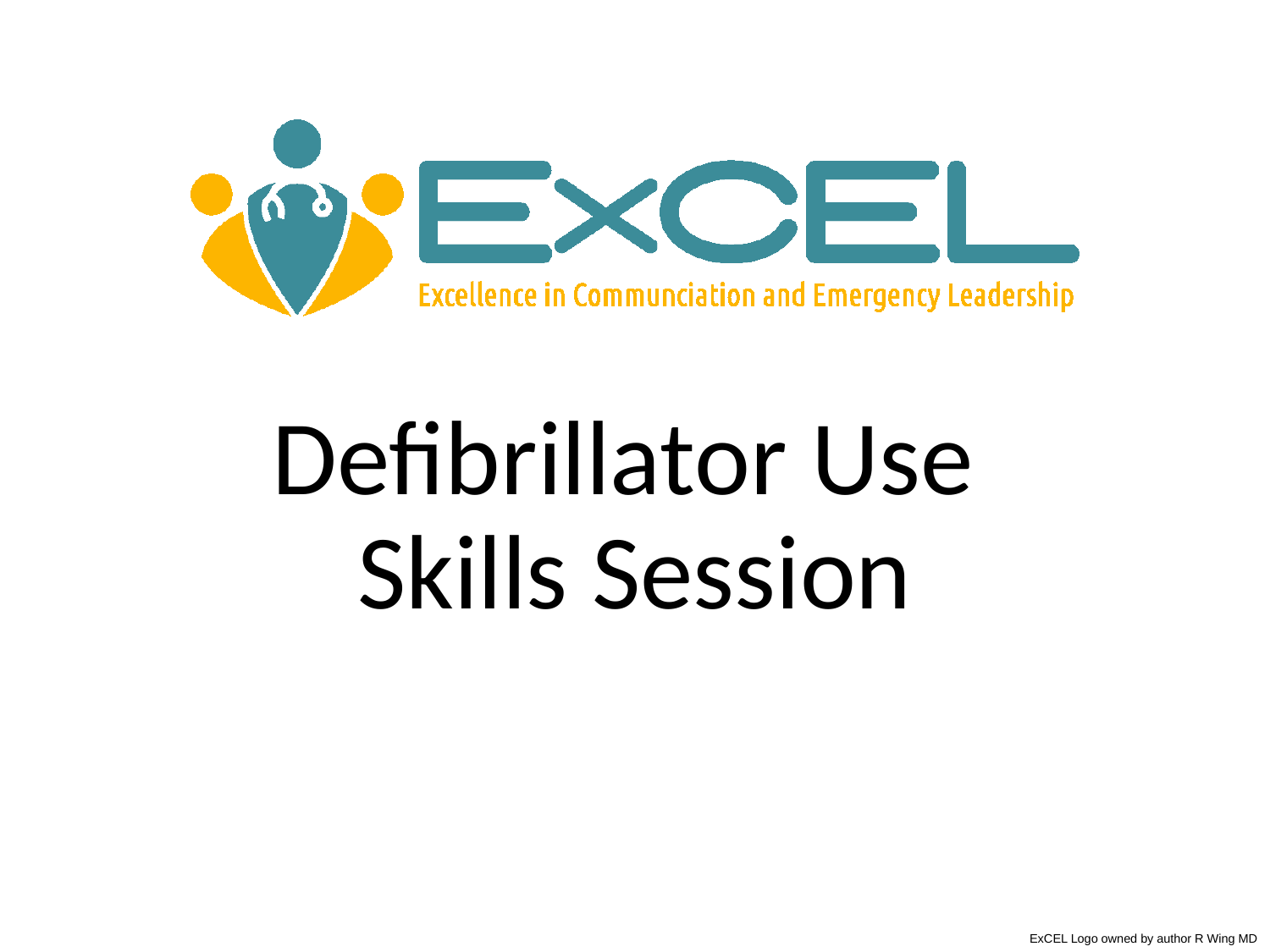

# Defibrillator Use Skills Session
ExCEL Logo owned by author R Wing MD

## Slide 2
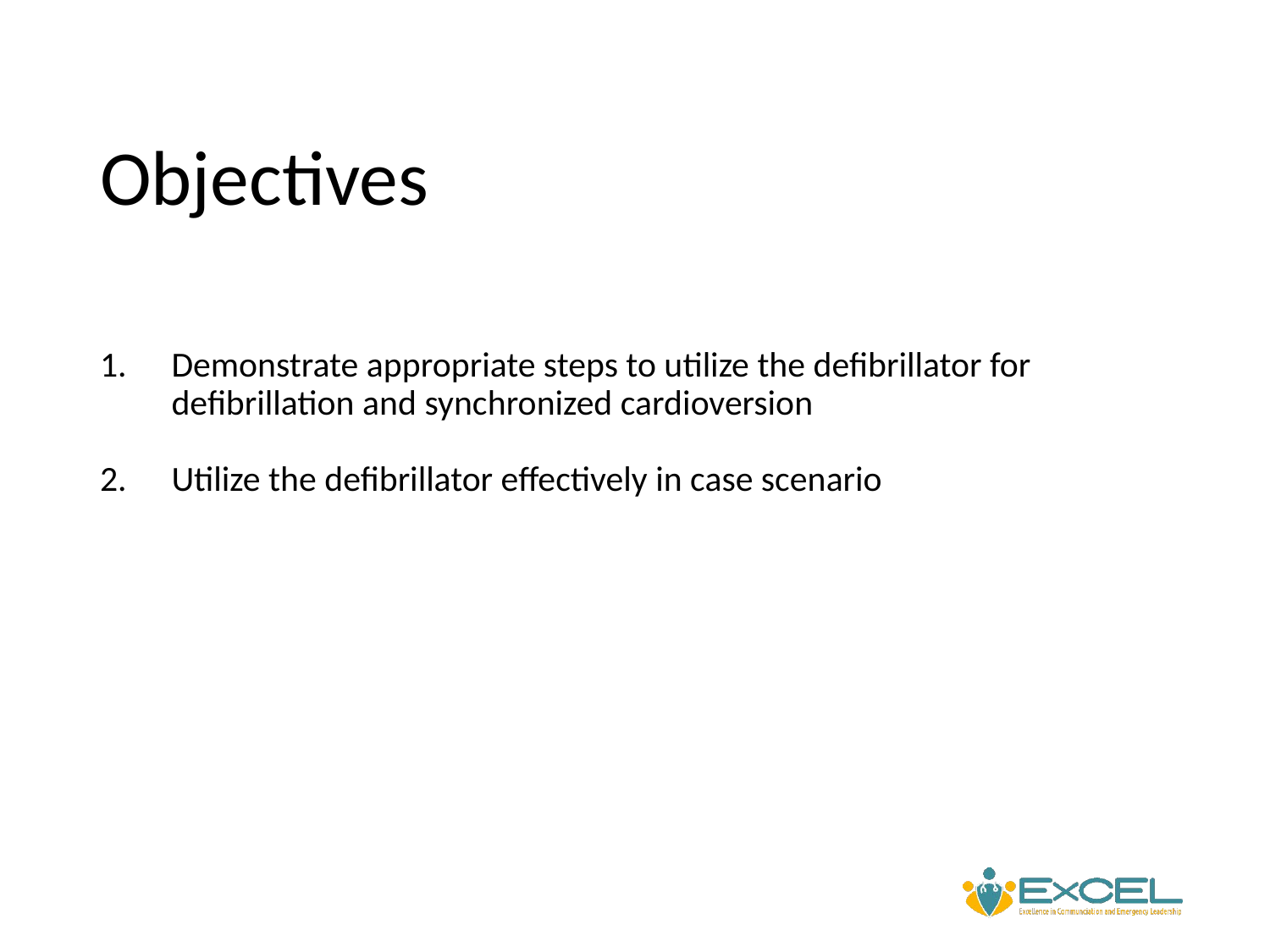

# Objectives
Demonstrate appropriate steps to utilize the defibrillator for defibrillation and synchronized cardioversion
Utilize the defibrillator effectively in case scenario

## Slide 3
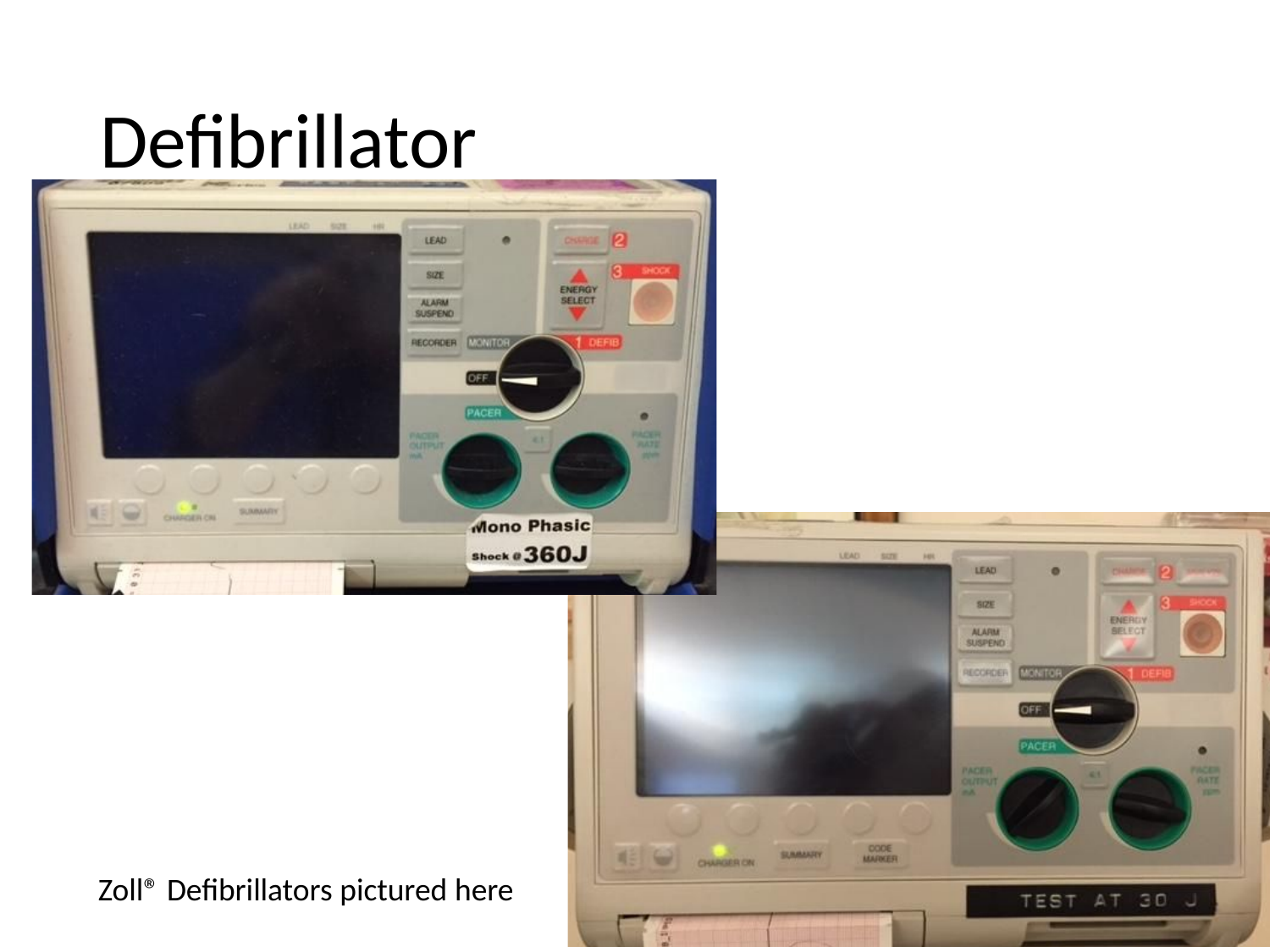

# Defibrillator
Zoll® Defibrillators pictured here

## Slide 4
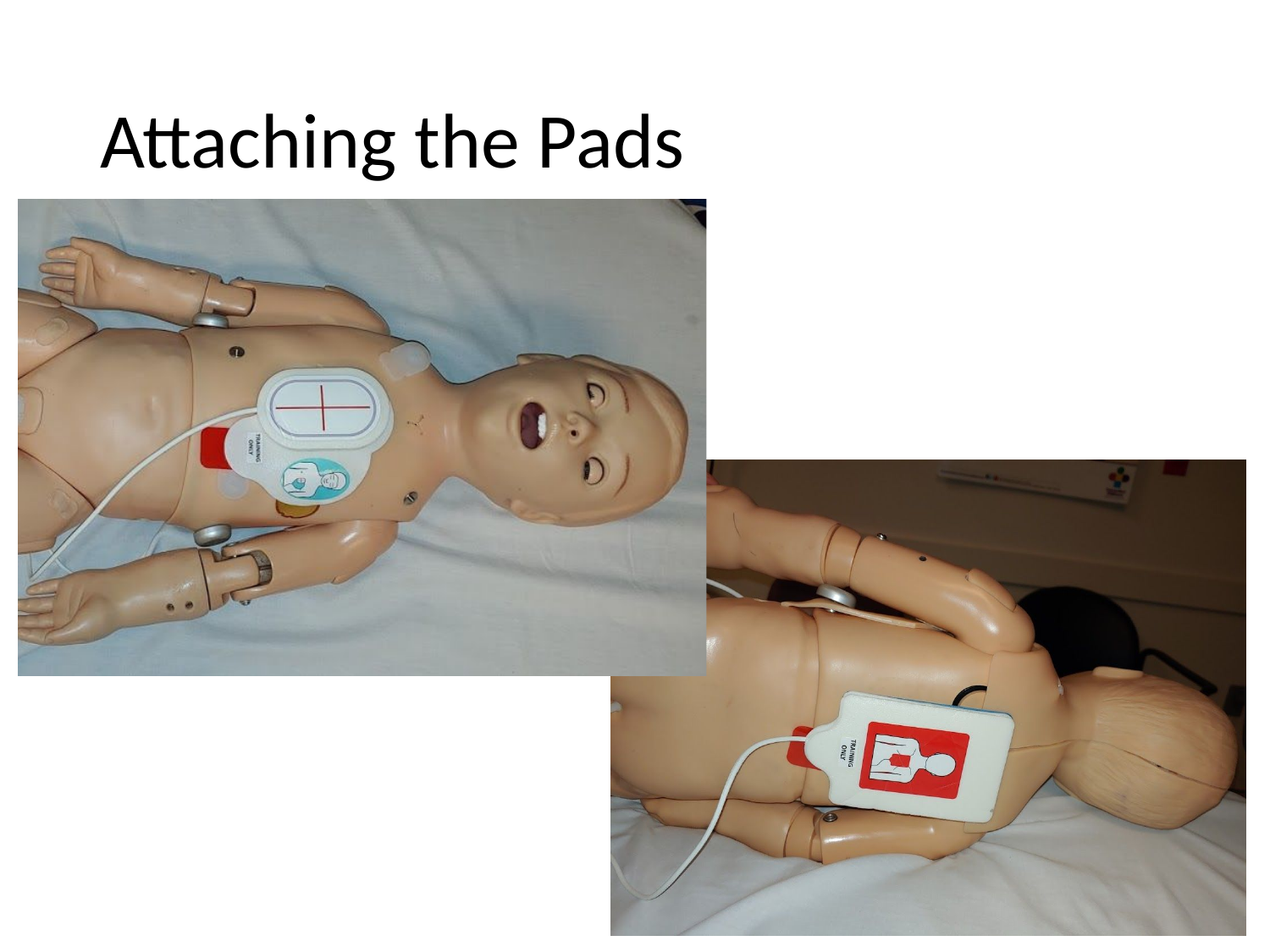

# Attaching the Pads

## Slide 5
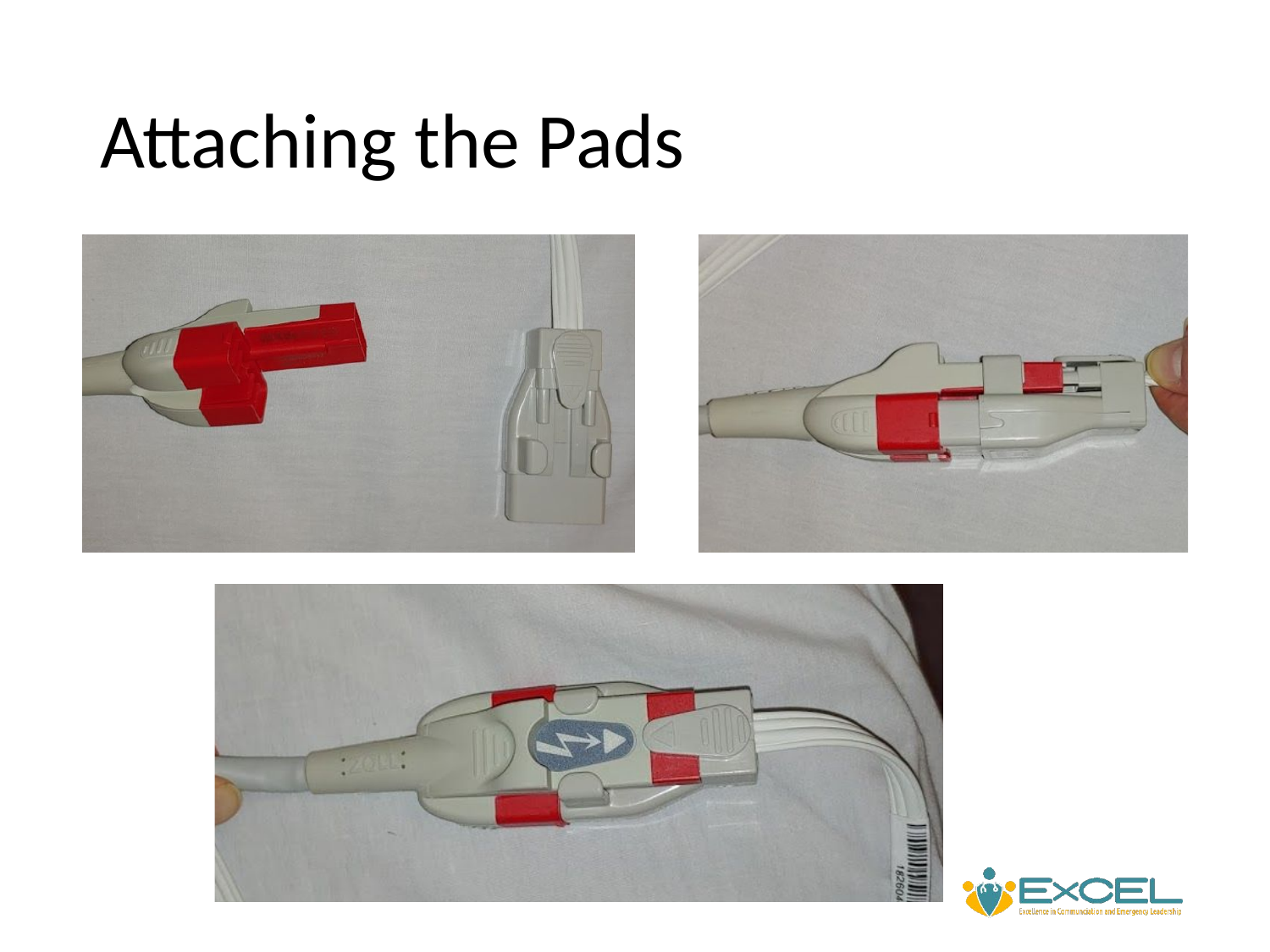

# Attaching the Pads

## Slide 6
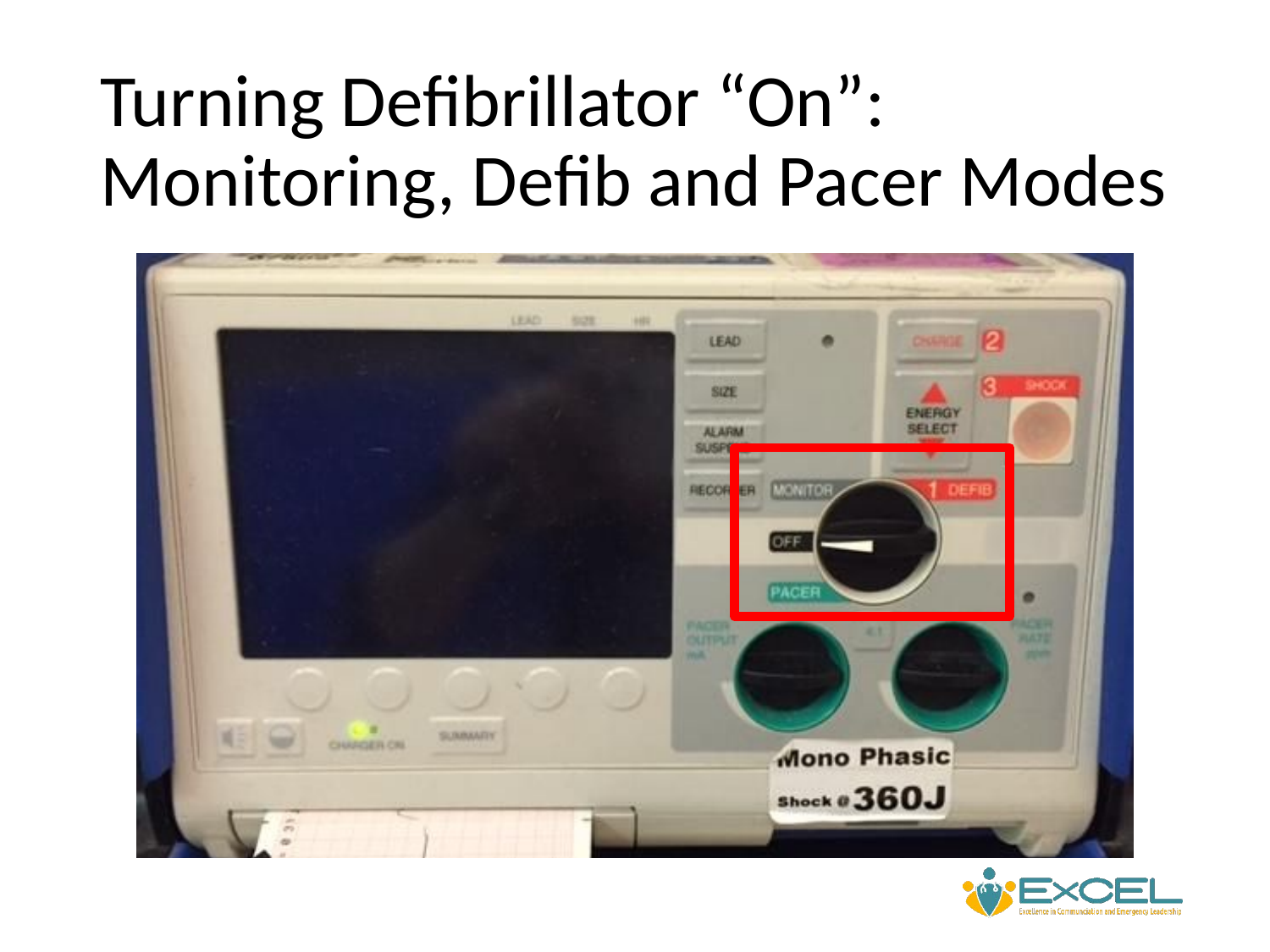

# Turning Defibrillator “On”: Monitoring, Defib and Pacer Modes

## Slide 7
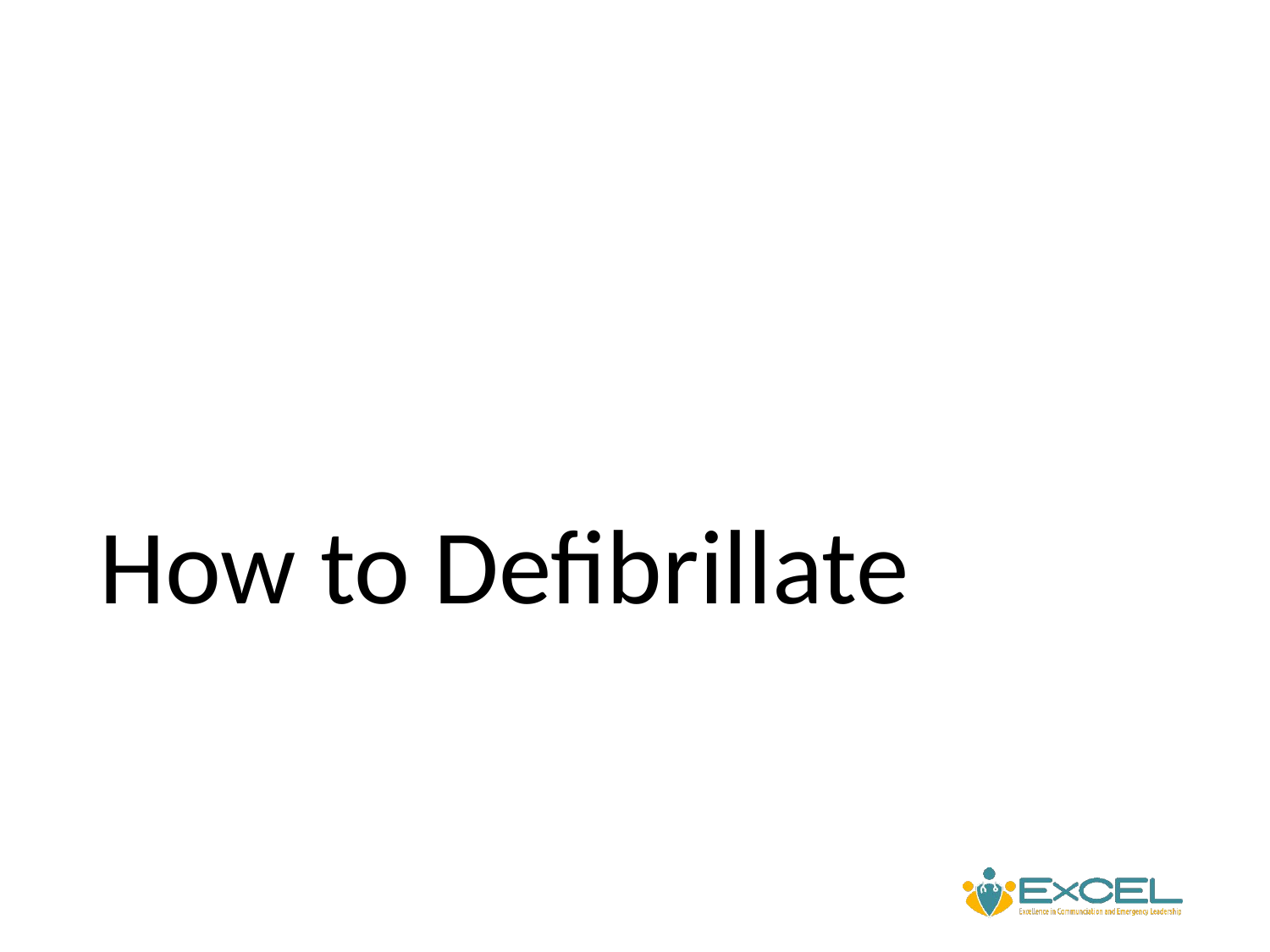

# How to Defibrillate

## Slide 8
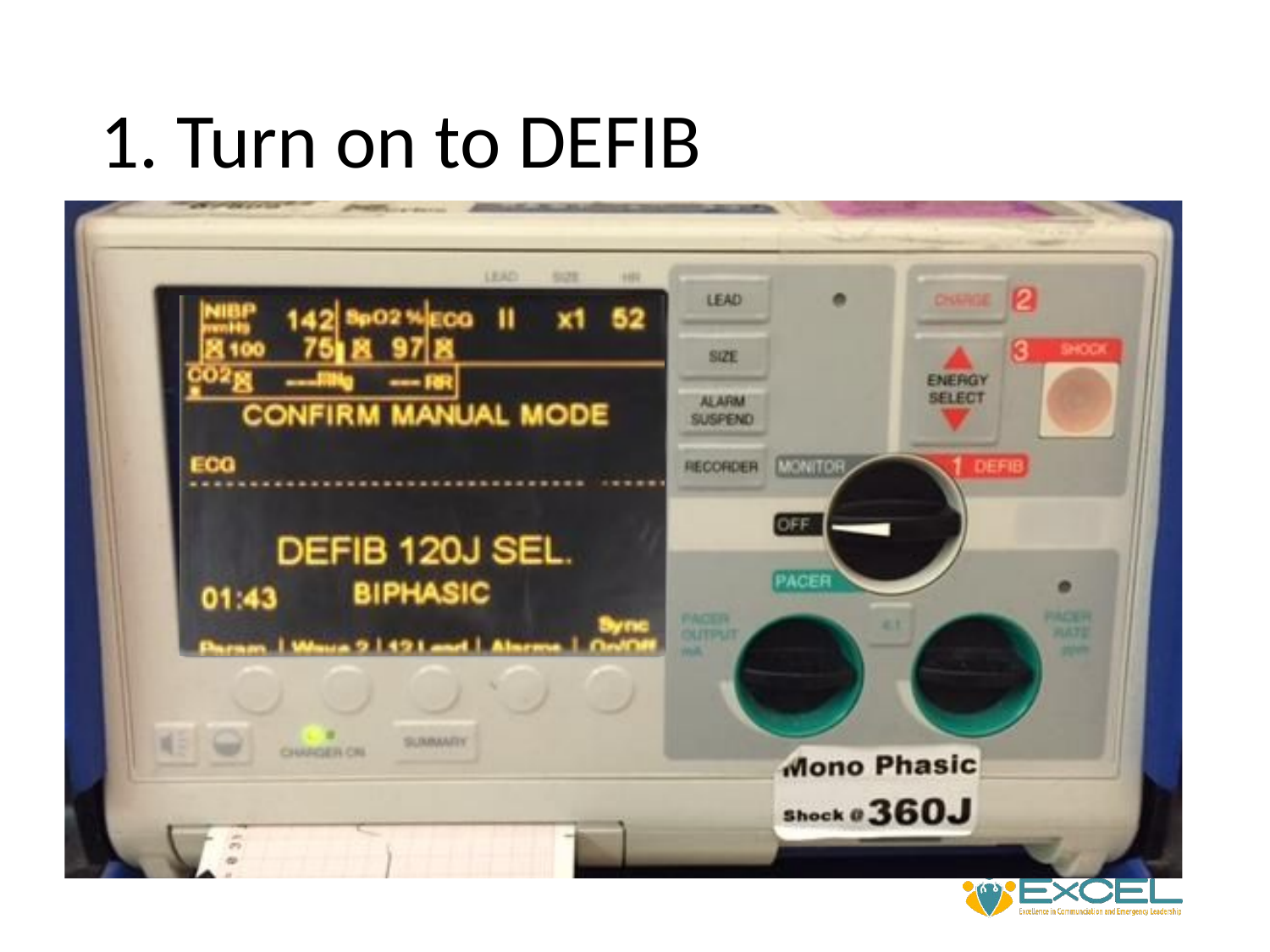

# 1. Turn on to DEFIB

## Slide 9
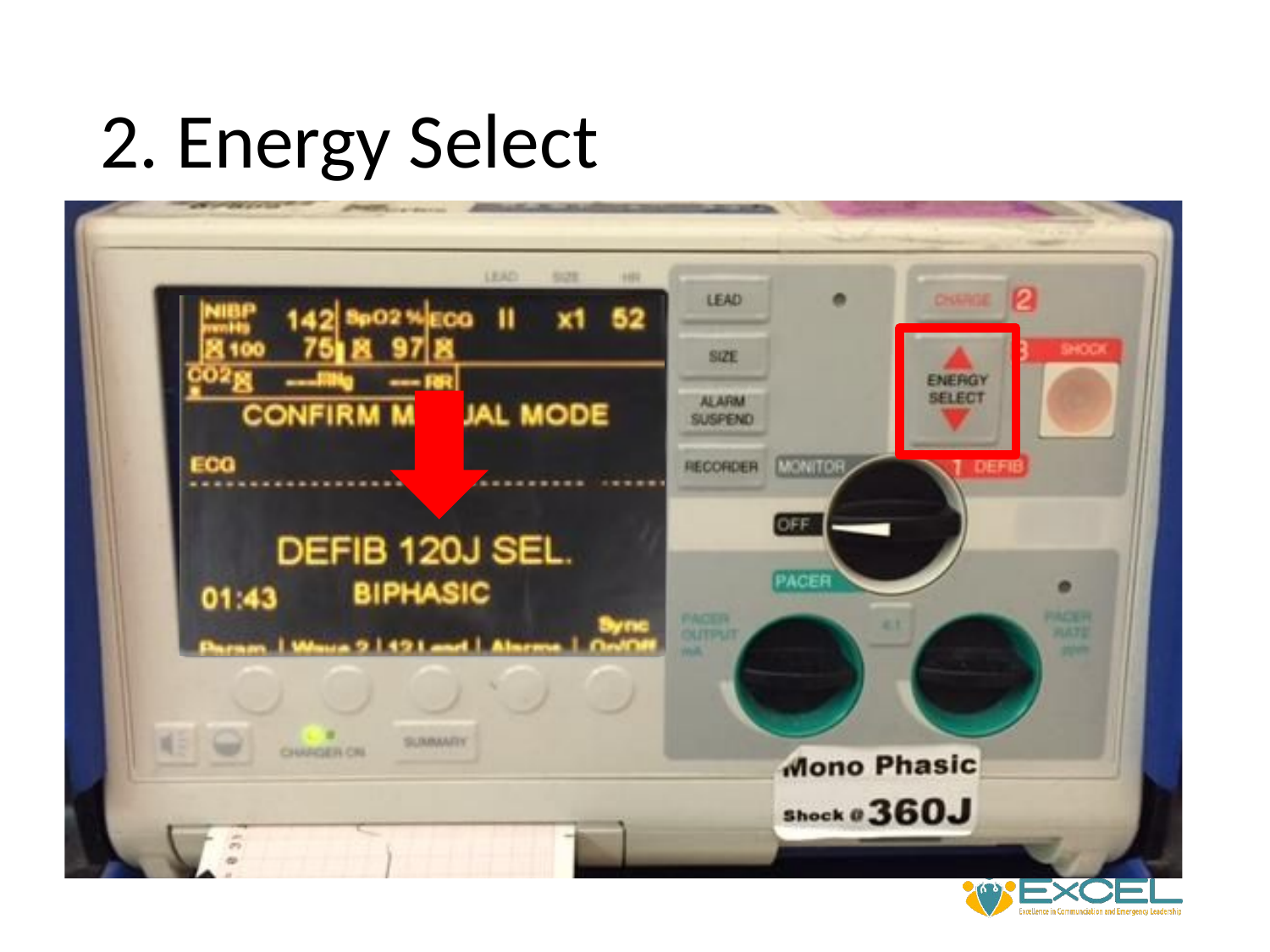

# 2. Energy Select

## Slide 10
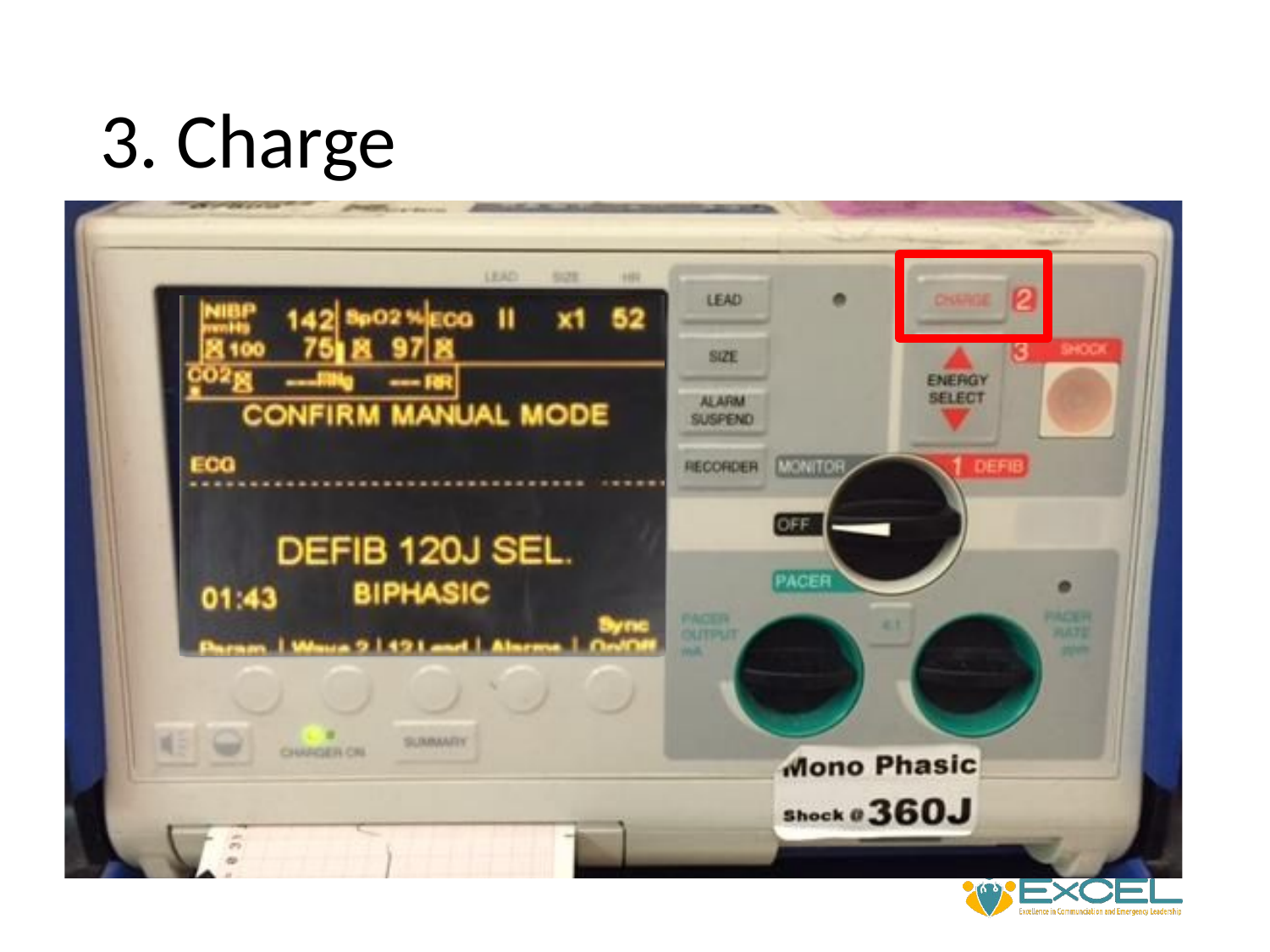

# 3. Charge

## Slide 11
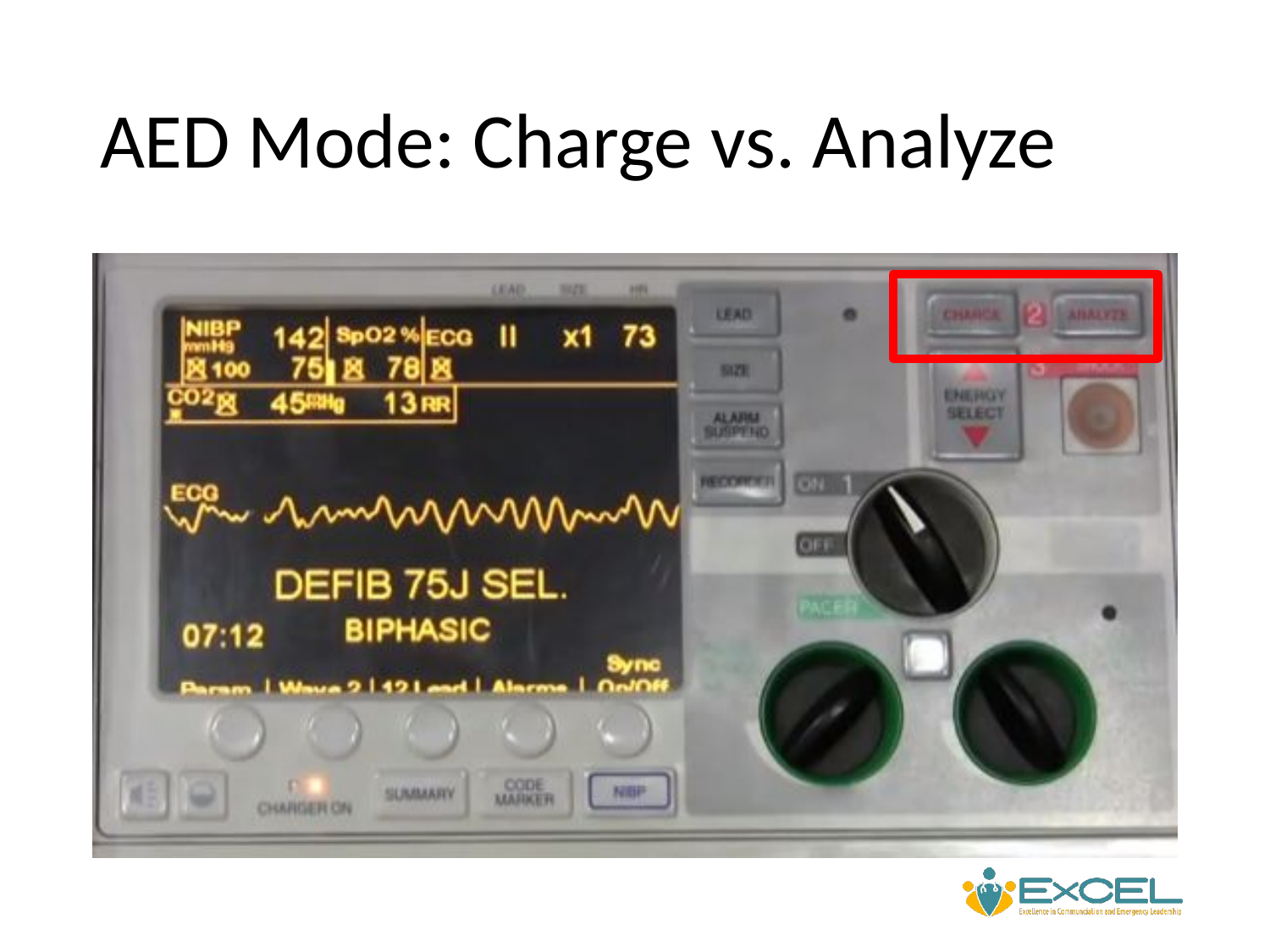

# AED Mode: Charge vs. Analyze

## Slide 12
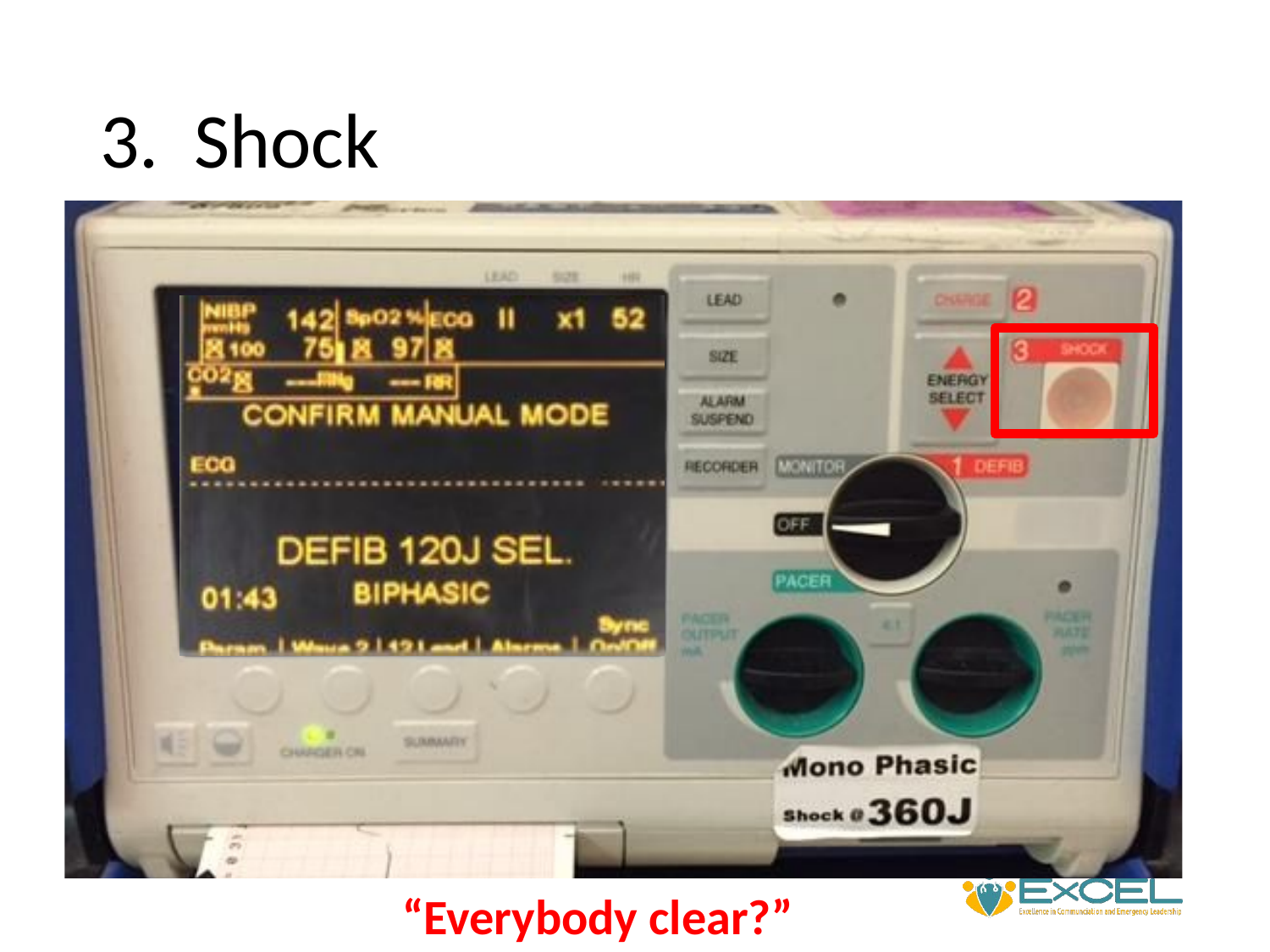

# 3. Shock
“Everybody clear?”

## Slide 13
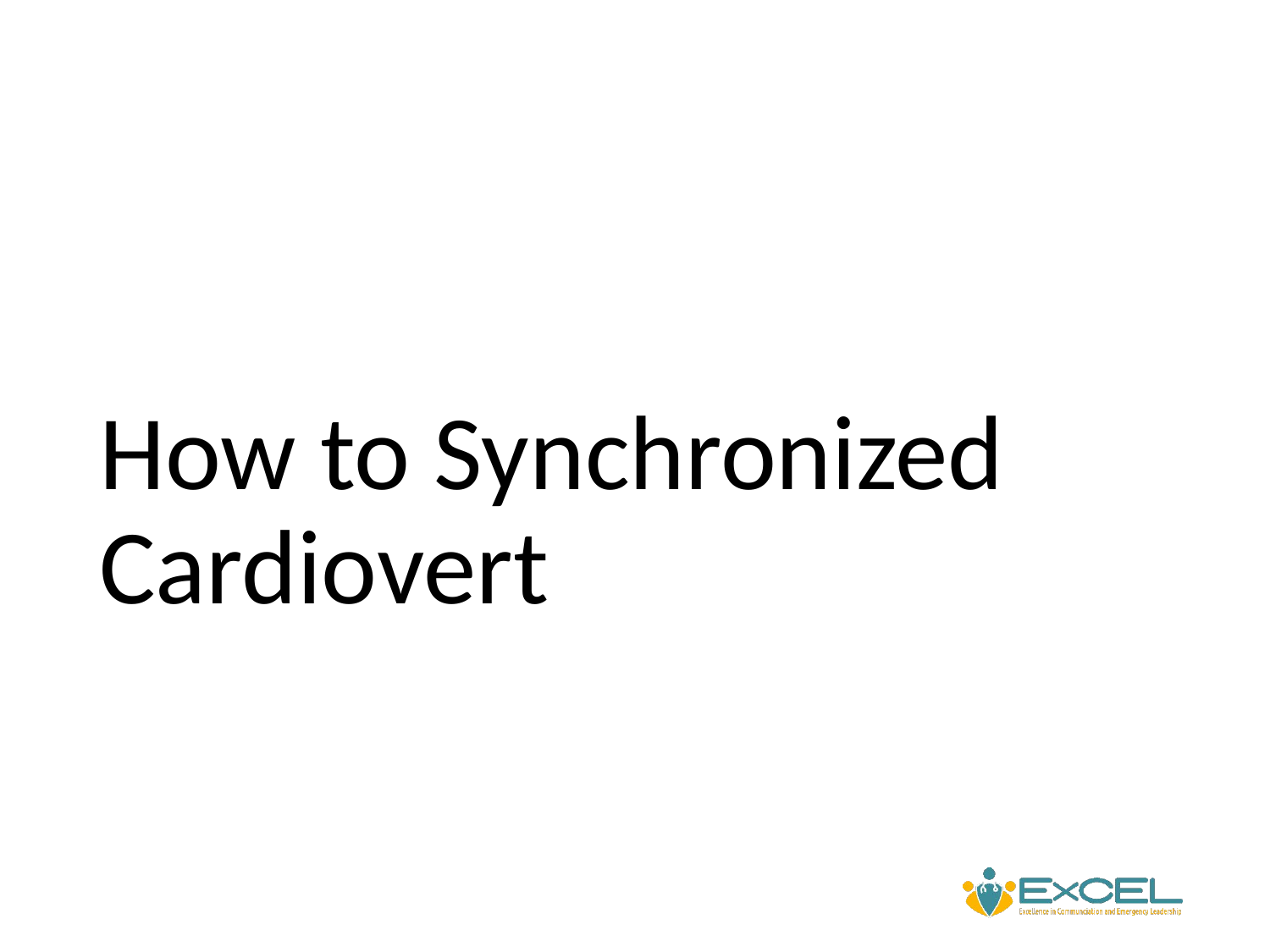

# How to Synchronized Cardiovert

## Slide 14
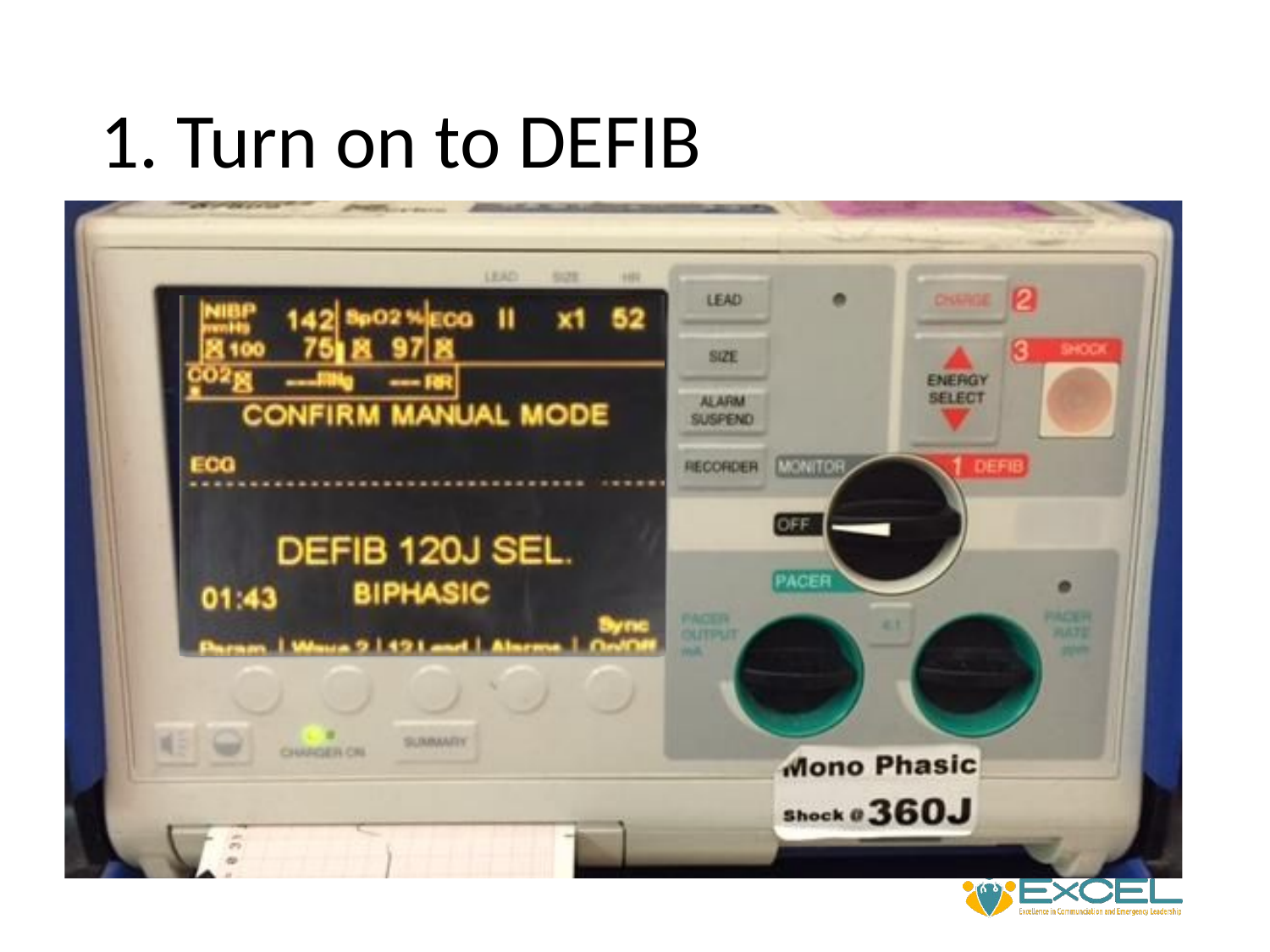

# 1. Turn on to DEFIB

## Slide 15
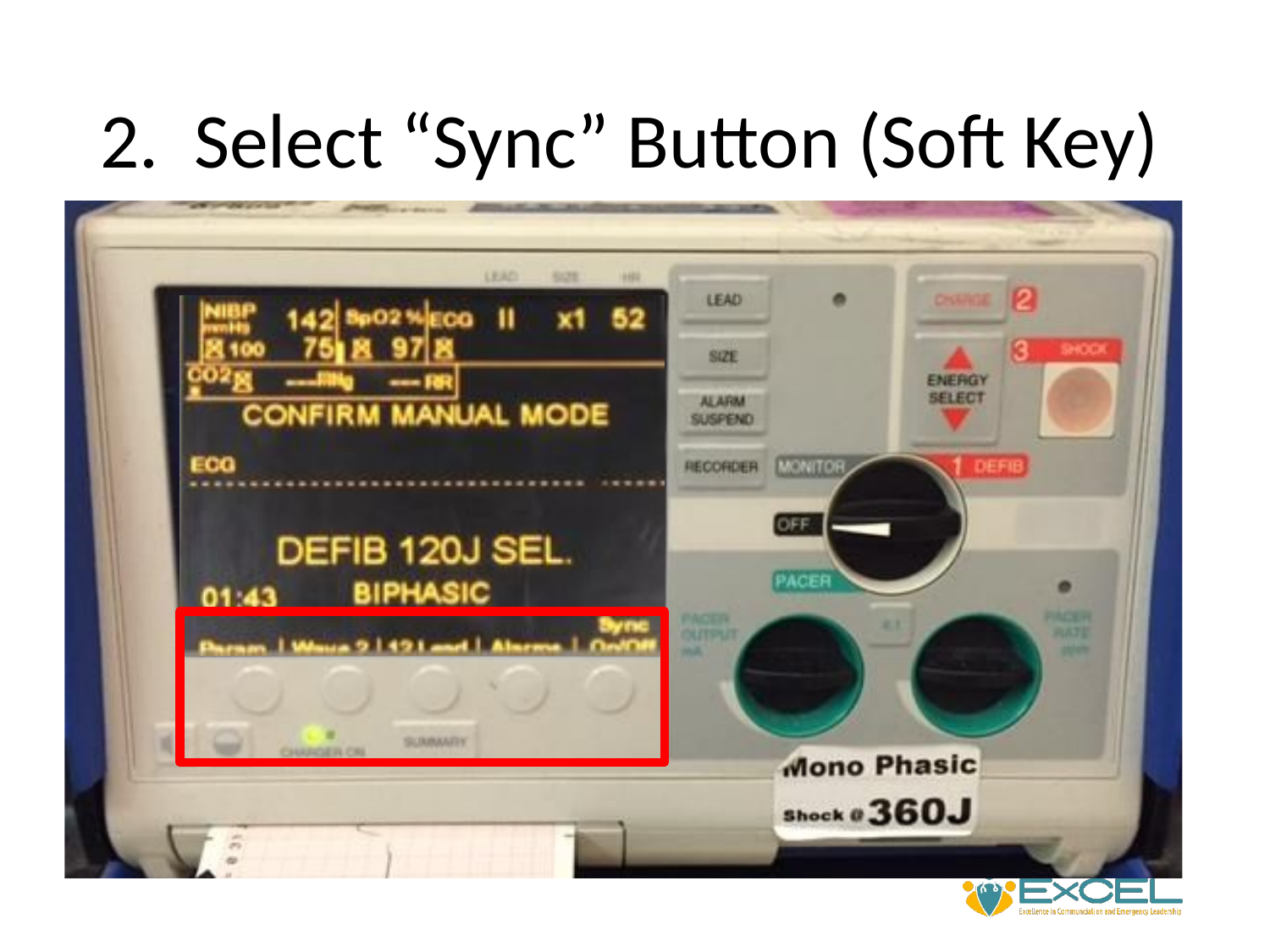

# 2. Select “Sync” Button (Soft Key)

## Slide 16
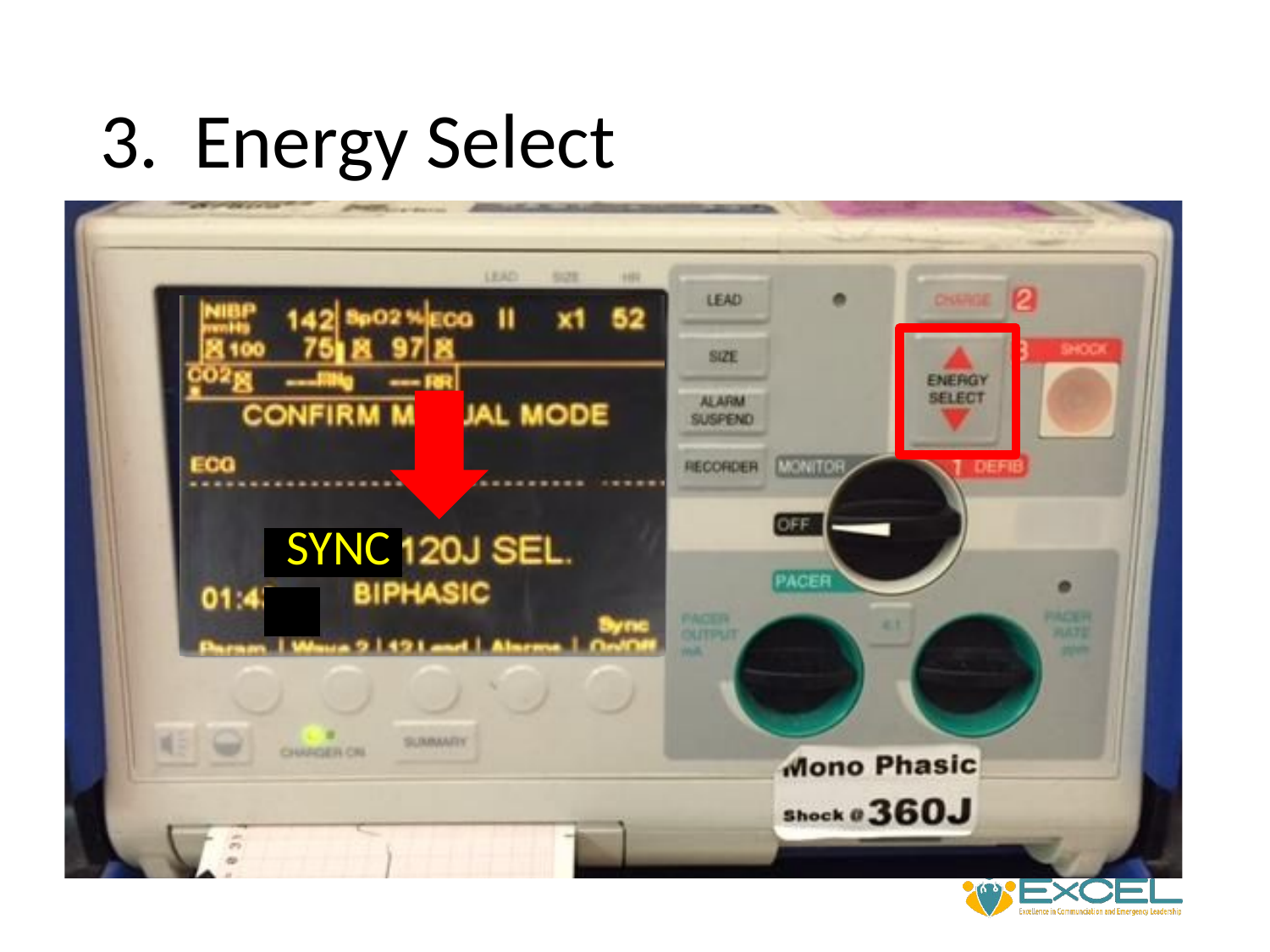

# 3. Energy Select
 SYNC

## Slide 17
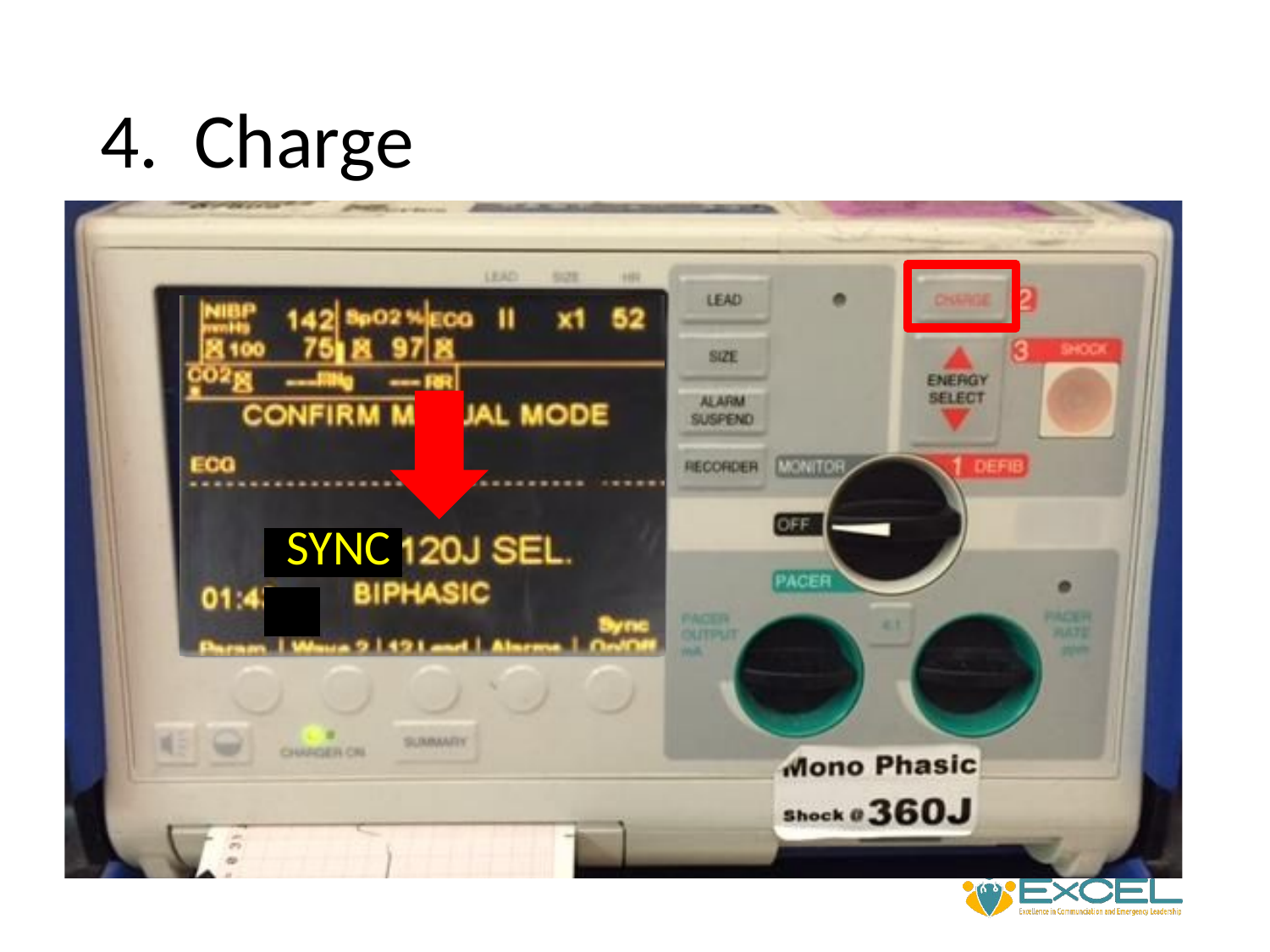

# 4. Charge
 SYNC

## Slide 18
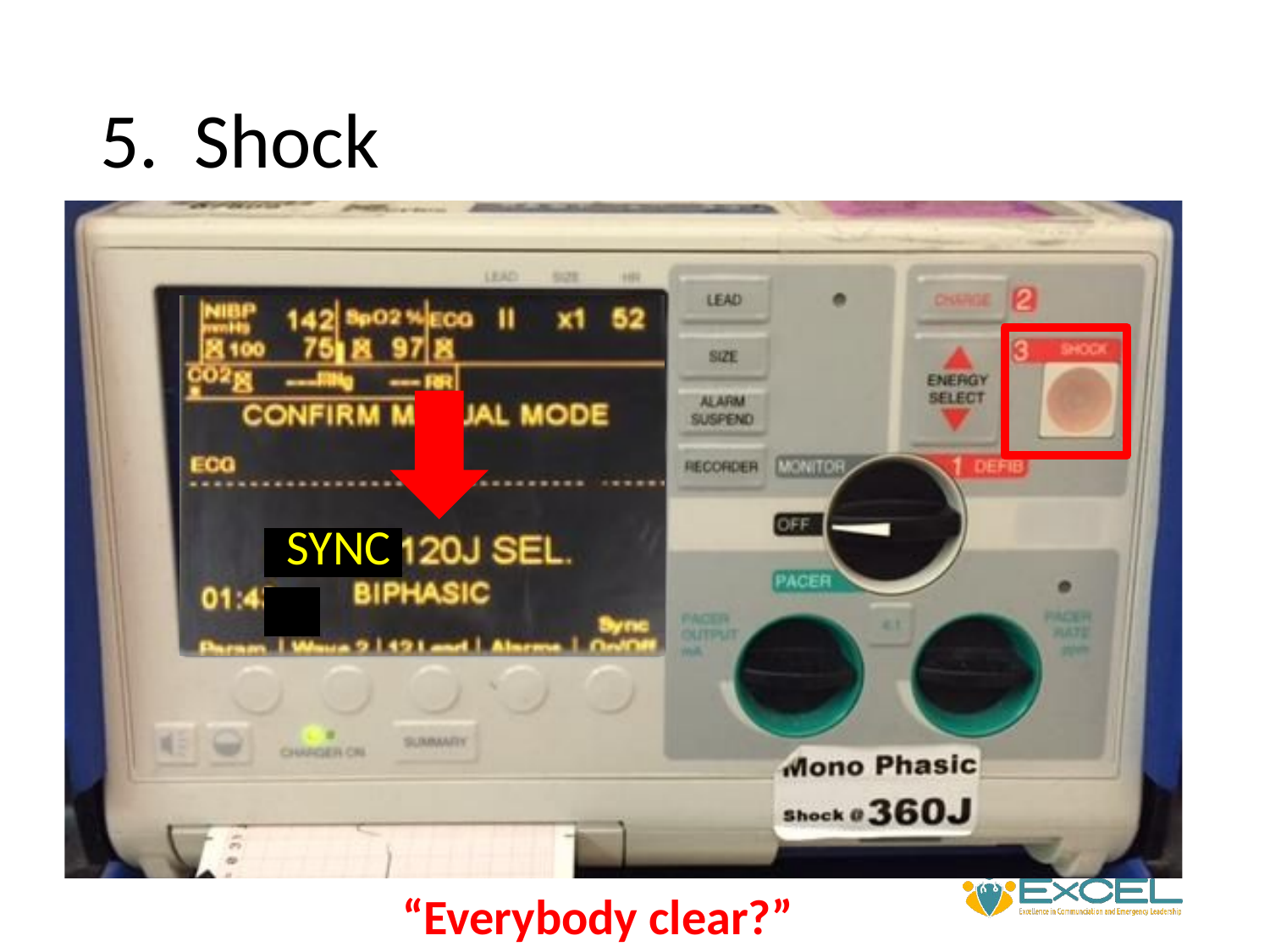

# 5. Shock
 SYNC
“Everybody clear?”

## Slide 19
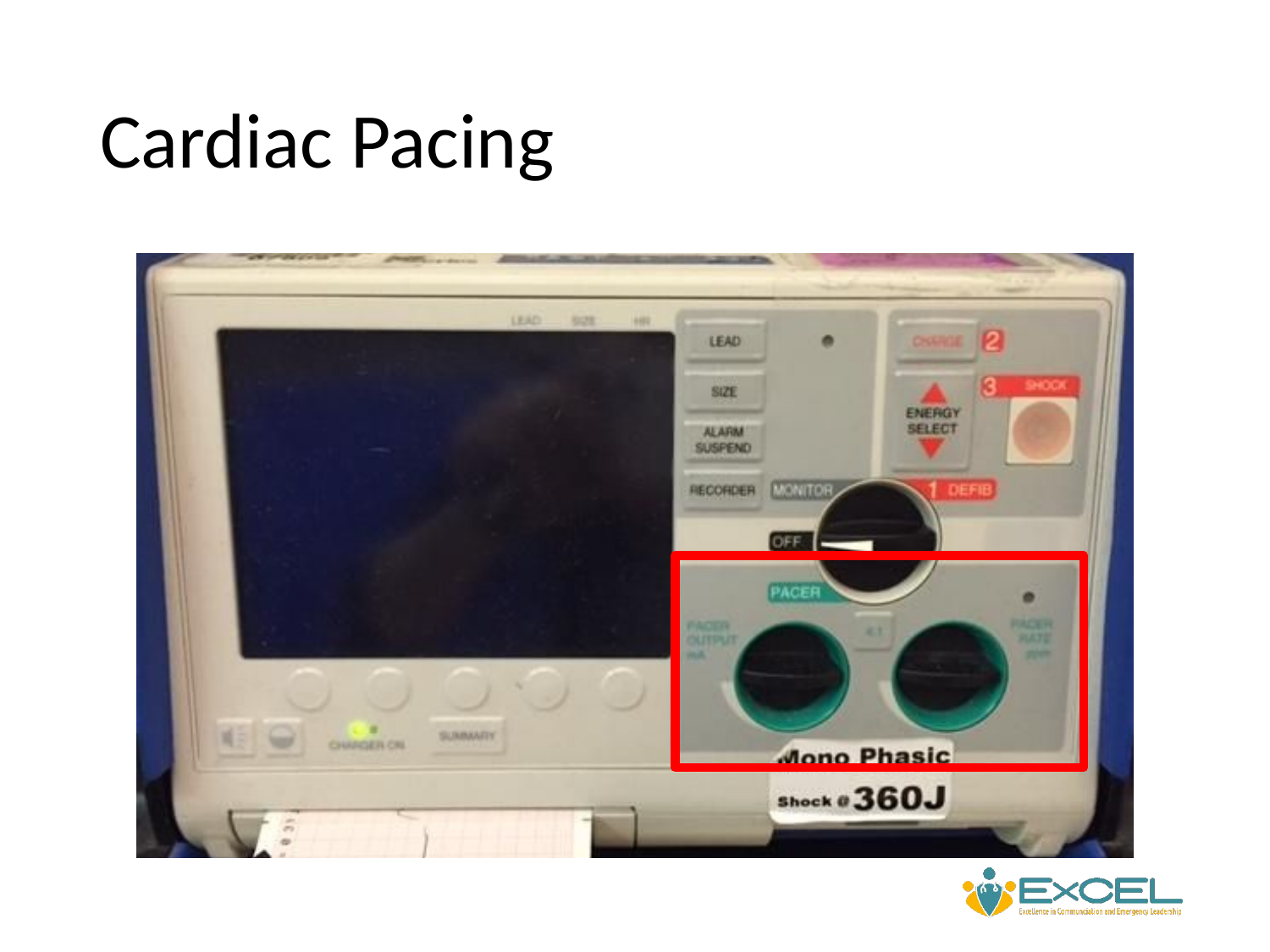

# Cardiac Pacing

## Slide 20
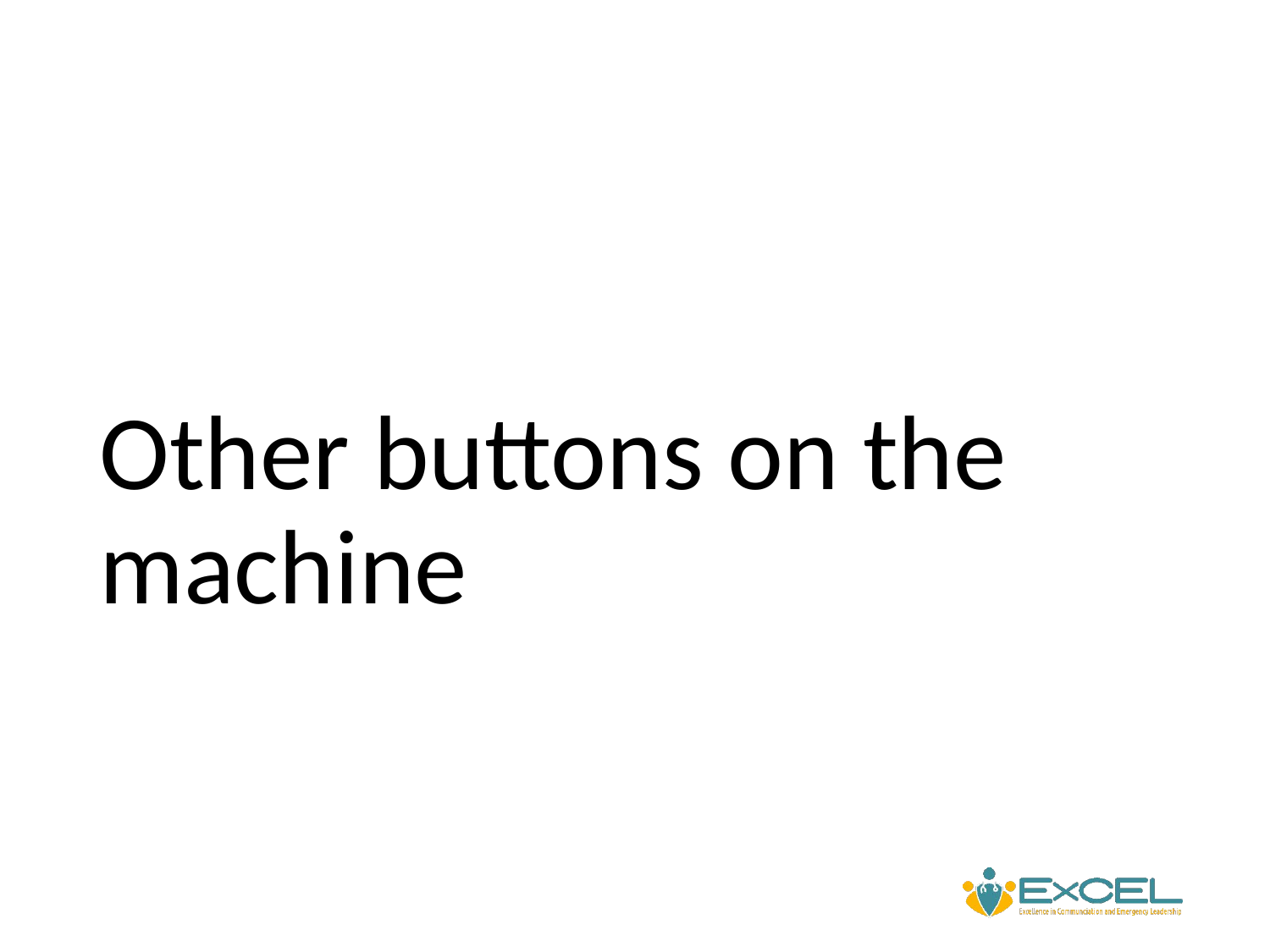

# Other buttons on the machine

## Slide 21
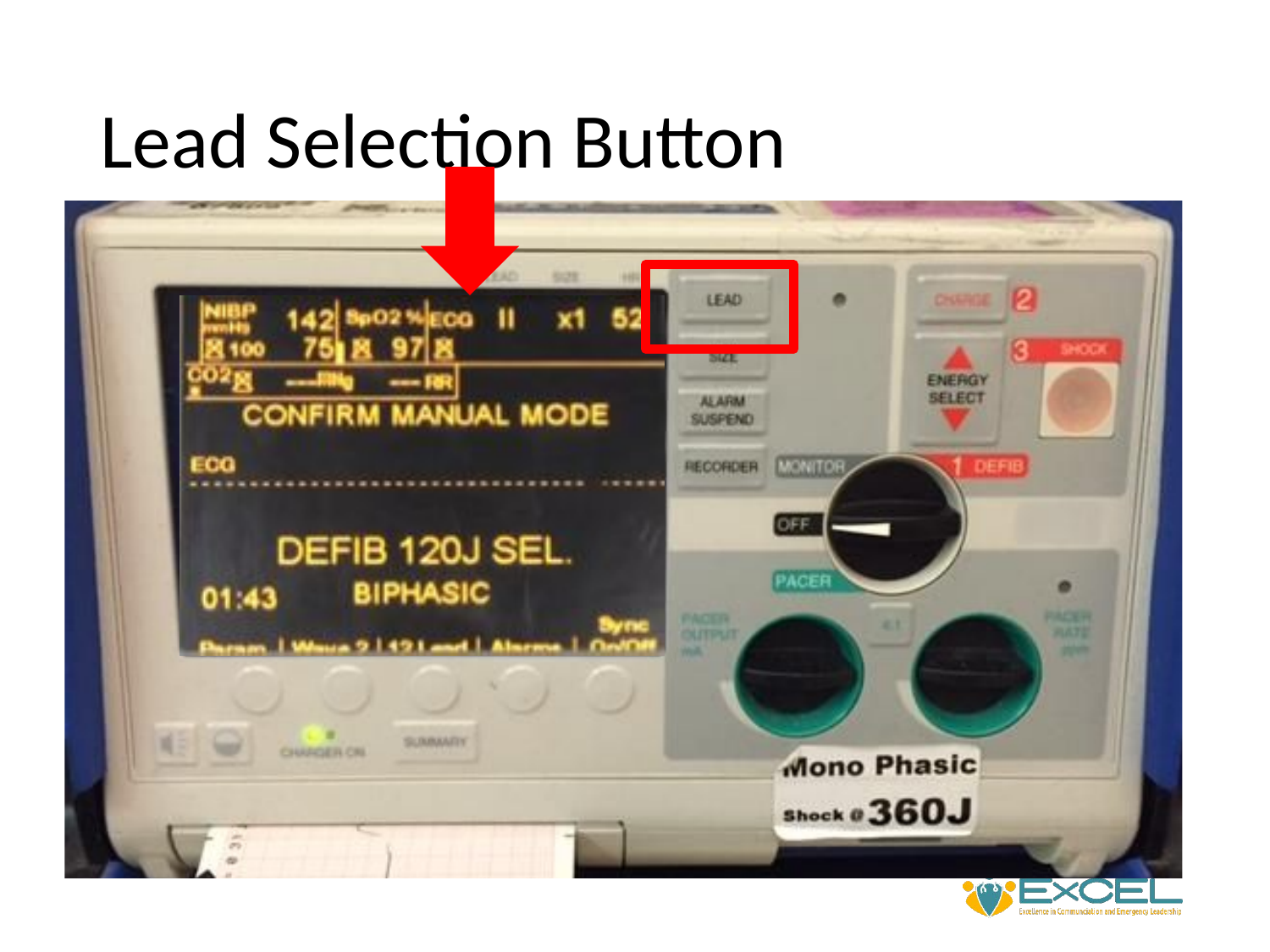

# Lead Selection Button

## Slide 22
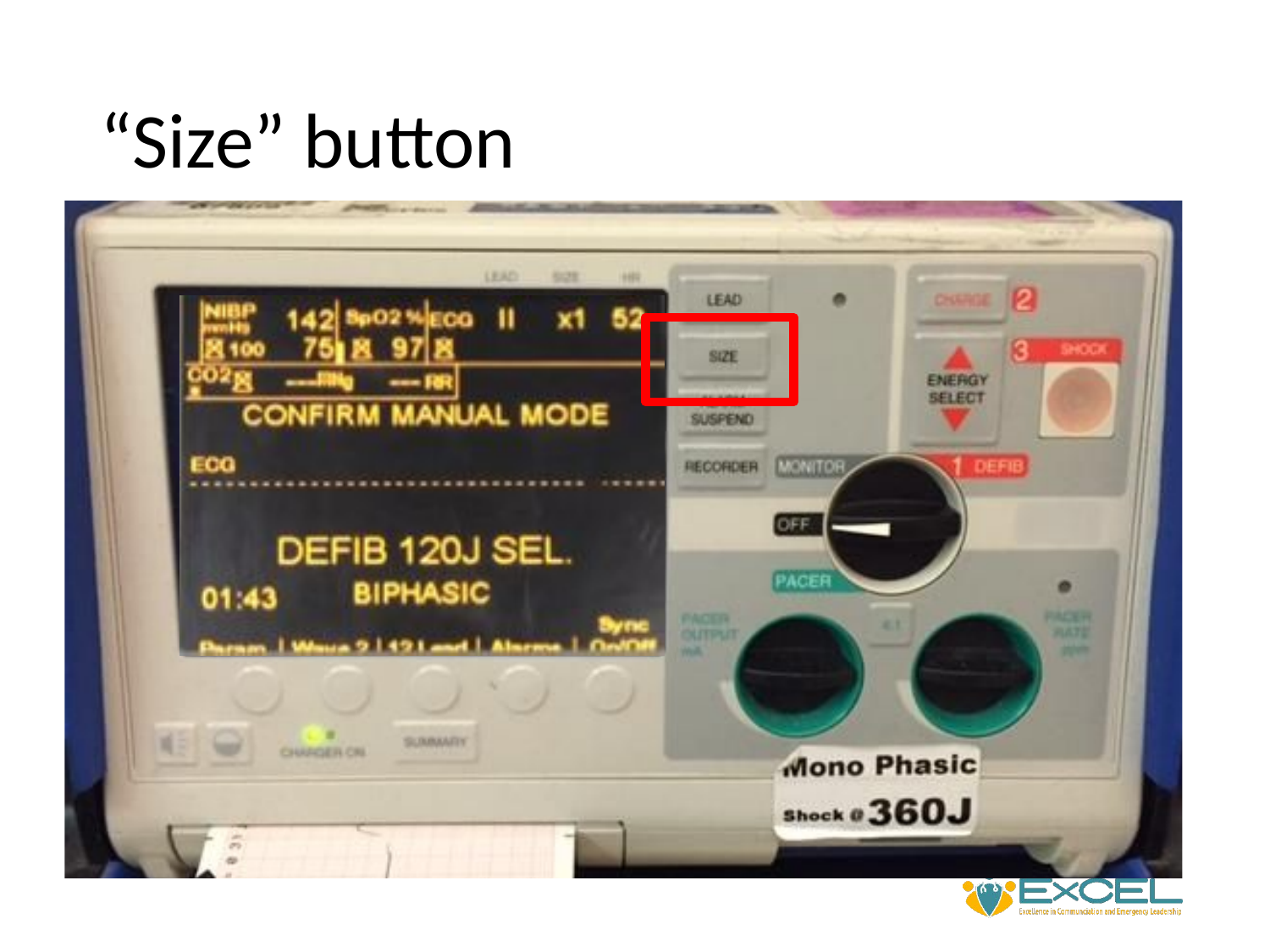

# “Size” button

## Slide 23
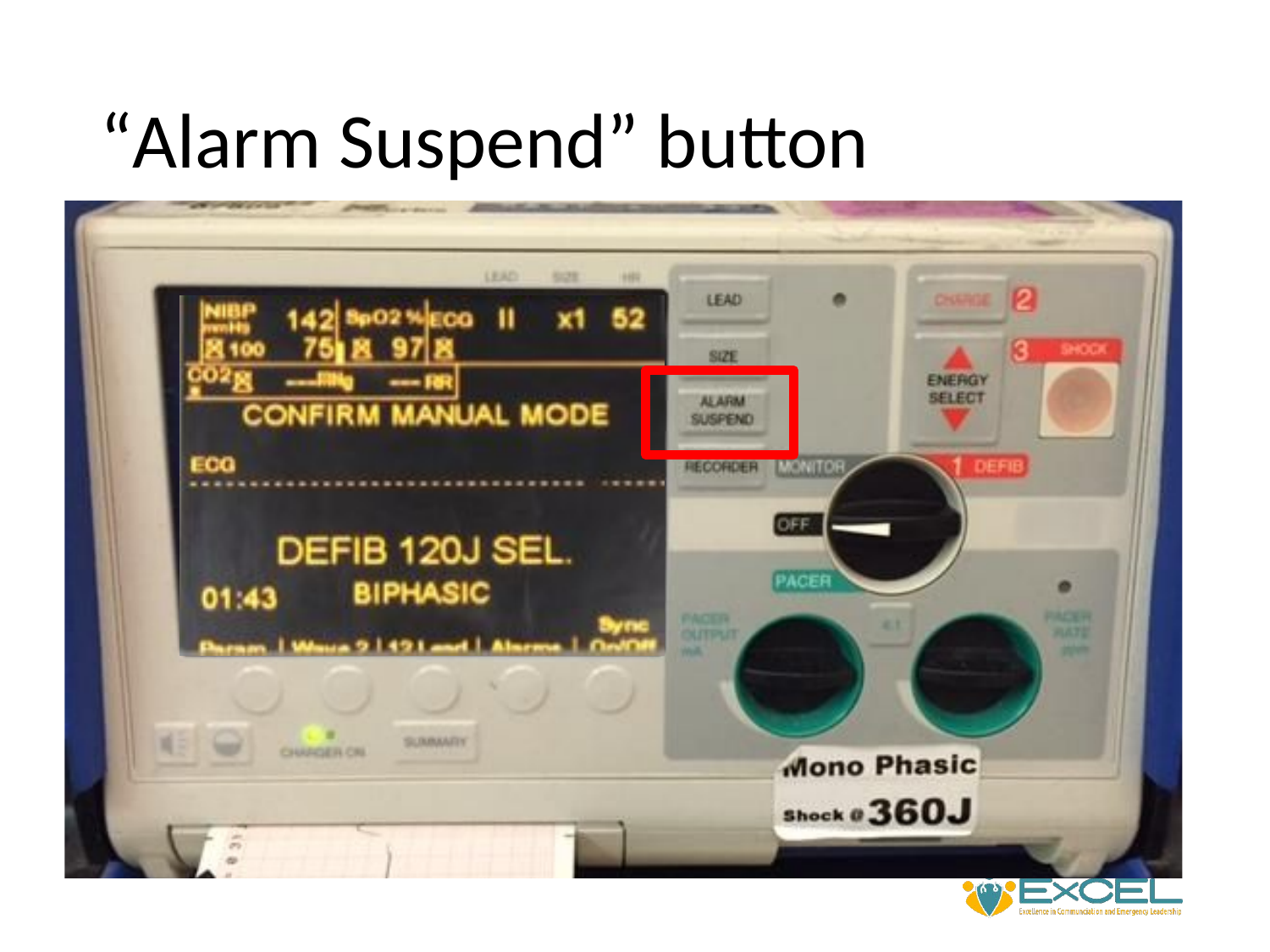

# “Alarm Suspend” button

## Slide 24
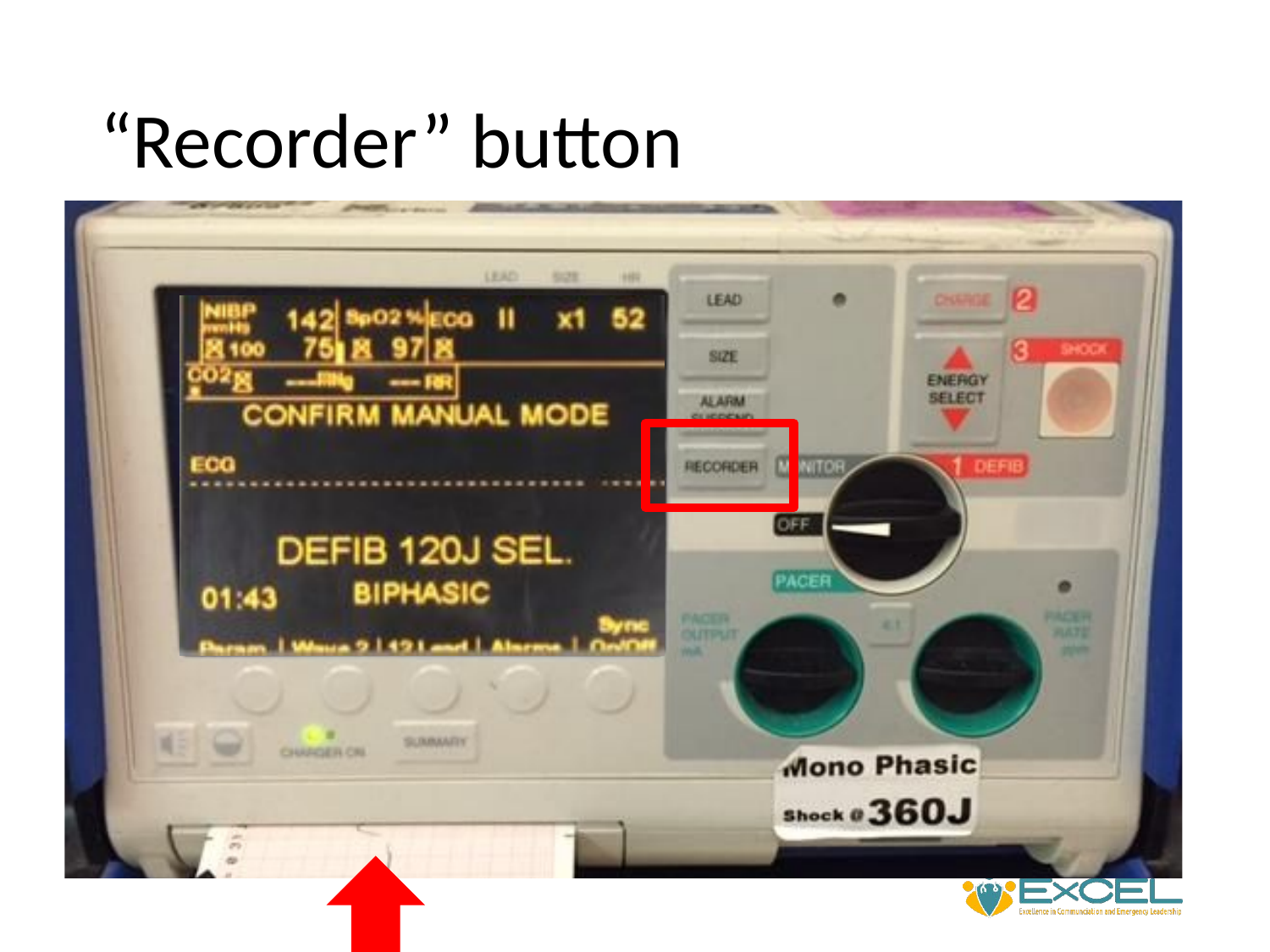

# “Recorder” button

## Slide 25
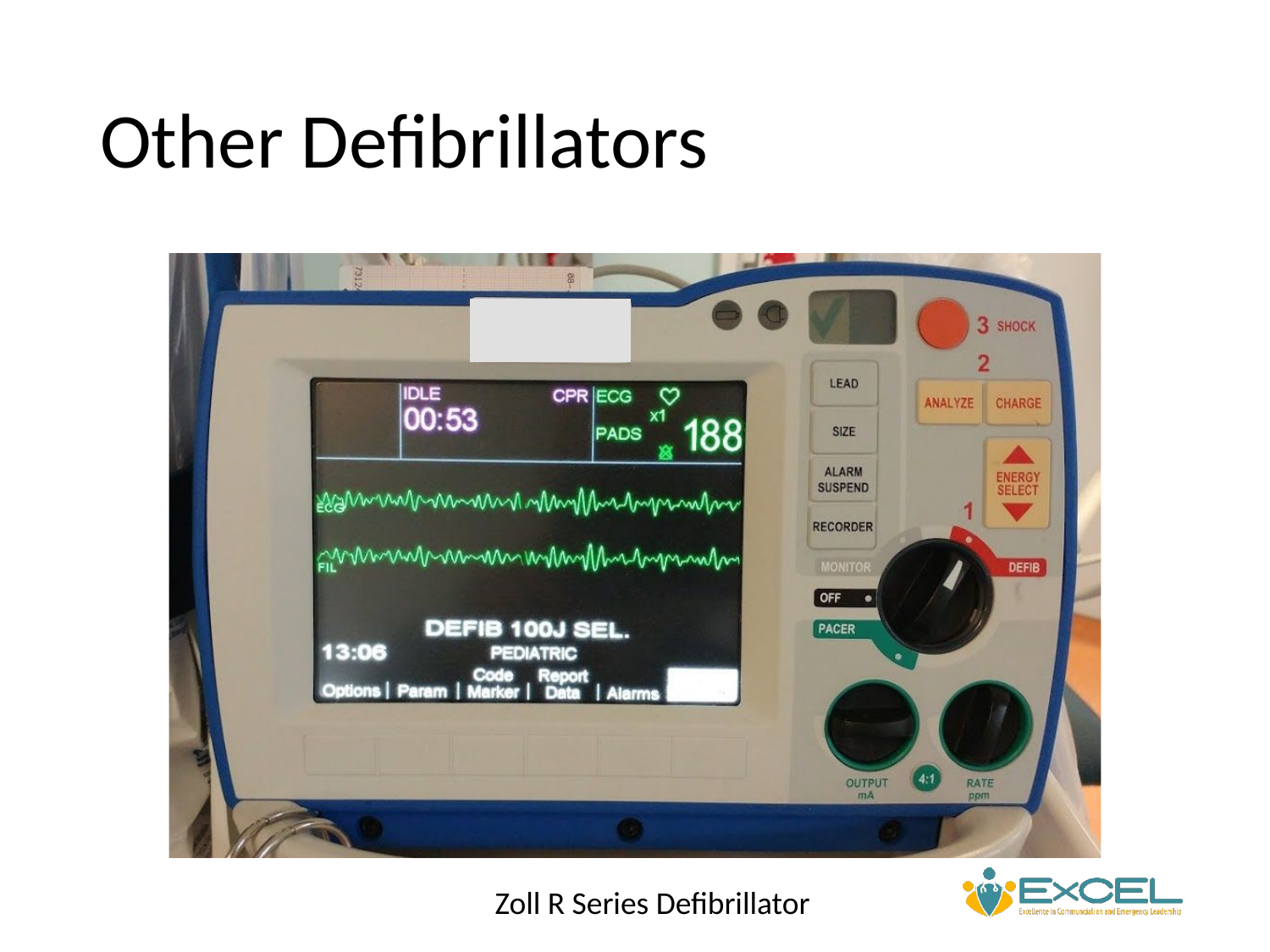

# Other Defibrillators
Zoll R Series Defibrillator

## Slide 26
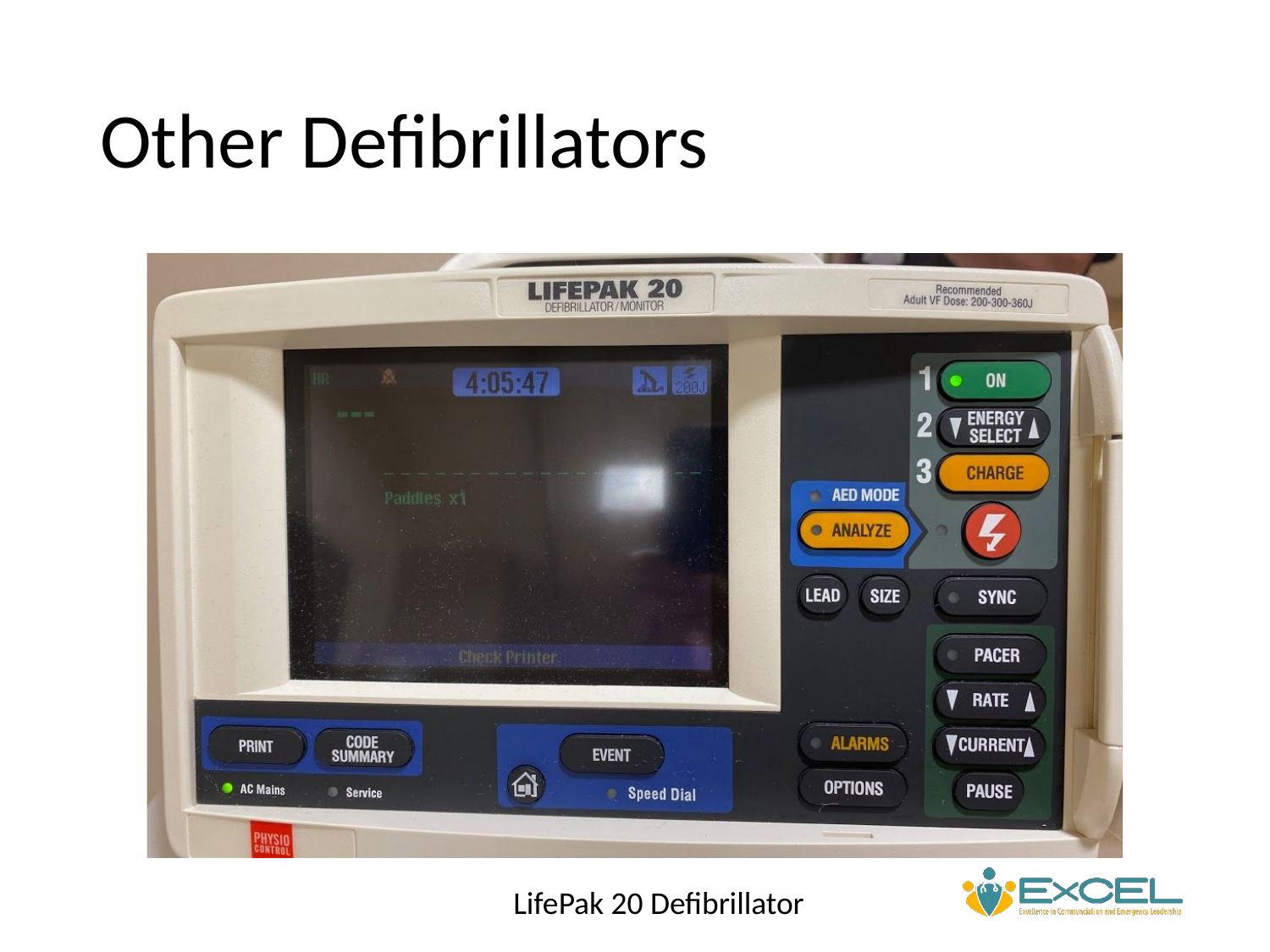

# Other Defibrillators
LifePak 20 Defibrillator
