# Supplementary material for: Excellence in Communication and Emergency Leadership (ExCEL): Pediatric Critical Care Resource Utilization Workshop for Residents
Source: MedEdPORTAL. 2022 Aug 16;18:11268. doi: 10.15766/mep_2374-8265.11268 (PMC9378690; doi:10.15766/mep_2374-8265.11268)
Supplement: Supplementary file 1 — Defibrillator Use Presentation.pptxCode Cart Skills Station.docxTransport Bag Skills Station.docxIntroduction to Defibrillator.docxDefibrillator Use Skills Station Cases.docxDefibrillator Use Skills Session Rhythm Strips.pptxExCEL Critical Care Workshop Surveys.docx [file mep_2374-8265.11268-s001.zip › B. Code Cart Skills Station.docx]

**Pediatric Code Cart Skills Session**

The following curriculum is meant to provide a standardized structure for review and subsequent hands-on scavenger hunt with an institution-specific pediatric code cart. Please note that the locations listed in the tables below may be different in different institutions. Facilitators should familiarize themselves with the code carts in their own institutions prior to facilitating this skills session.

1. **Pediatric Code Cart Review:** Instructors should provide a “tour” of the pediatric code cart, pointing out where critical care items are located. This is easiest done with a top-down approach, starting with the top of the cart and working down the cart, pulling out each drawer and physically pulling out items for participants to see. If critical items such as a backboard and step stool are not included in the code cart, the location of those items should also be reviewed with participants.
2. **Case-Based Scavenger Hunt:** Divide residents into small groups (2-3 residents) and present a clinical scenario to the team. Residents should find necessary equipment to care for their mock patient in the pediatric code cart.

**Case #1**

Scenario: You are on the wards and it is 2AM when the code pager goes off. You arrive moments later to find the nurse standing by the patient’s bedside. The patient is an 8-year-old surgical patient whom you do not know. The nurse tells you that she found the patient unresponsive. At this same moment, another nurse brings the code cart to the room. Both of the nurses that are with you are new and are not familiar with the code cart.

| **Action** | **Necessary Critical Materials** | **Location (Institution-Specific)** |
| --- | --- | --- |
| 1. Check a pulse (NO pulse) |  |  |
| 2. Start chest compressions | Back Board  Step Stool | Back of cart  Side of cart |
| 3. Start BVM and coordinate CPR 15:2 | Appropriately-sized BVM | Bottom Drawer (“First Five Minutes Drawer”) |
| 4. Place patient on defibrillator (Note: Verbalize “PEA”) | Defibrillator  Pads  Consider monitor leads  Consider pulse ox | Top of cart  Bottom Drawer (“First Five Minutes Drawer”) |
| 5. Estimate Patient Weight | Broselow Tape | Bottom Drawer (“First Five Minutes Drawer”) |
| 6. Obtain Access | IV  Tape  Tegaderm  Extension tubing  IO  Flush | Access Drawer  Brought by PICU team |
| 7. Administer Epinephrine | Code Dose Epinephrine | Medication Tray |
| 8. Check a pulse (+ pulse) → End Scenario |  |  |

**Case #2**

Scenario: It’s 5AM and you are a resident on the wards. The code pager goes off directing you to the room of a 13-month-old with bronchiolitis on HFNC that you admitted earlier in the night. The nurse tells you that she found the patient unresponsive with SpO_2_ 88% on 8L HFNC, 100% FiO_2_.

| **Action** | **Necessary Critical Materials** | **Location (Institution-Specific)** |
| --- | --- | --- |
| 1. Check a pulse (NO pulse) |  |  |
| 2. Start chest compressions | Back Board  Step Stool | Back of cart  Side of cart |
| 3. Start BVM and coordinate CPR 15:2 | Appropriately-sized BVM | Bottom Drawer (“First Five Minutes Drawer”) |
| 4. Place patient on defibrillator (Note: Verbalize “PEA”) | Defibrillator  Pads  Consider monitor leads  Consider pulse ox | Top of cart  Bottom Drawer (“First Five Minutes Drawer”) |
| 5. Estimate Patient Weight | Broselow Tape | Bottom Drawer (“First Five Minutes Drawer”) |
| 6. Obtain Access | IV  Tape  Tegaderm  Extension tubing  IO  Flush | Access Drawer  Brought by PICU team |
| 7. Administer Epinephrine | Code Dose Epinephrine | Medication Tray in Medication Drawer |
| 8. Prepare for definitive airway | Intubation Tray  Consider LMA | Intubation Drawer |
| 8. Check a pulse (+ pulse) → End Scenario |  |  |
