# Supplementary material for: Excellence in Communication and Emergency Leadership (ExCEL): Pediatric Critical Care Resource Utilization Workshop for Residents
Source: MedEdPORTAL. 2022 Aug 16;18:11268. doi: 10.15766/mep_2374-8265.11268 (PMC9378690; doi:10.15766/mep_2374-8265.11268)
Supplement: Supplementary file 1 — Defibrillator Use Presentation.pptxCode Cart Skills Station.docxTransport Bag Skills Station.docxIntroduction to Defibrillator.docxDefibrillator Use Skills Station Cases.docxDefibrillator Use Skills Session Rhythm Strips.pptxExCEL Critical Care Workshop Surveys.docx [file mep_2374-8265.11268-s001.zip › C. Transport Bag Skills Station.docx]

**Pediatric Transport Bag Skills Session**

The following curriculum is meant to provide a standardized structure for review and subsequent hands-on scavenger hunt with an institution-specific pediatric transport bag. Please note that the locations listed in the tables below may be different in different institutions. Facilitators should familiarize themselves with the code carts in their own institutions prior to facilitating this skills session.

1. **Pediatric Transport Bag Review:** Instructors should provide a “tour” of the pediatric transport bag, pointing out where critical care items are located. Instructors should physically pull out items for participants to see. If critical items are not included in the transport bag, the location of those items should also be reviewed with participants.
2. **Case-Based Scavenger Hunt:** Divide residents into small groups (2-3 residents) and present a clinical scenario to the team. Residents should find necessary equipment to care for their mock patient in the pediatric transport bag.

**Case #1**

Scenario: It’s 4:45PM and you are the ED resident preparing to leave for the day. You have one patient left in the ED, a 5-year-old with a known seizure disorder who presented in status epilepticus, requiring 2 doses of lorazepam and a levetiracetam load which stopped his seizures. Due to his prolonged post-ictal period and continued altered mental status, you decide to admit him to the PICU.

His nurse tells you that his bed is ready in the PICU and asks you if you are ready to travel with them.

| **Prompt** | **Anticipated Action** | **Necessary Critical Materials** |
| --- | --- | --- |
| *“What do you want to bring with you?”* |  | Transport Bag  Anti-Epileptic drug (AED, ex. benzodiazepine), appropriately dosed |
| *“The elevator gets stuck with you inside! While you wait for facilities, you witness another seizure. Generalized tonic-clonic, now going on 5 minutes.”* | Give AED (ex. lorazepam) | Anti-Epileptic Drug  IV flush |
| *“After 1 dose of AED, his seizure stops. You notice that his oxygen saturation is decreasing down to 85%. What are your next steps?”* | Reposition patient’s airway |  |
| *“Oxygen saturation is down to 83% and the respiratory rate is about 4.”* | Start Bagging  Consider Airway Adjunct | Ambu Bag  Appropriate-sized Mask  NPA |
| *“Sats improve to 98% with bagging and NPA in place.”* | Case End |  |

**Case #2**

Scenario: It’s 3AM and you are the resident in the ED. You have just intubated a 6-month-old patient for acute respiratory failure of unclear etiology and it’s time to bring the patient to the MRI scanner. You are traveling with a nurse, a tech, and a respiratory therapist.

| **Prompt** | **Anticipated Action** | **Necessary Critical Materials** |
| --- | --- | --- |
| *“What do you want to bring with you?”* |  | Transport Bag  Sedation med (appropriately-dosed)  Paralytic med (appropriately-dosed) |
| *“While in the elevator, the patient begins to move and cough. You realize that he has self-extubated as his O_2_ sats are dropping rapidly and you no longer have an end-tidal reading. What do you do?”* | Start to bag-mask ventilate the patient | Ambu Bag  Appropriate-sized Mask  NPA and/or OPA |
| *“You notice that you are bagging effectively as O_2_ sats return to 96%. You are now waiting in the MRI scanner and you have been informed that he needs to be re-intubated prior to his scan. Your attending is on the way to intubate there and asks you to have everything ready. What do you do?”* | Prepare intubation equipment | Intubation Checklist  Cuffed ETT (3.5)  Stylet  Miller 1 blade  End-tidal CO_2_ detector  Ambu Bag  Tape |
| *“Your nurse notes that the patient’s IV is no longer working. She asks if she needs to go back to the ED or if we brought supplies.”* | Get IV equipment | IV needle  Extension tubing  NS Flush  Tegaderm |
| *“Your nurse places the IV and your attending successfully intubates the patient. The patient is able to successfully get the MRI done and is transported without incident to the PICU.“* | Case End |  |
