# Supplementary material for: Excellence in Communication and Emergency Leadership (ExCEL): Pediatric Critical Care Resource Utilization Workshop for Residents
Source: MedEdPORTAL. 2022 Aug 16;18:11268. doi: 10.15766/mep_2374-8265.11268 (PMC9378690; doi:10.15766/mep_2374-8265.11268)
Supplement: Supplementary file 1 — Defibrillator Use Presentation.pptxCode Cart Skills Station.docxTransport Bag Skills Station.docxIntroduction to Defibrillator.docxDefibrillator Use Skills Station Cases.docxDefibrillator Use Skills Session Rhythm Strips.pptxExCEL Critical Care Workshop Surveys.docx [file mep_2374-8265.11268-s001.zip › D. Introduction to Defibrillator.docx]

**Defibrillator Use – Introduction to Defibrillator**

The following curriculum is meant for use by instructors to provide a standardized structure for review and practice of defibrillation and cardioversion.

Please note: different institutions may use different defibrillators. Facilitators must familiarize themselves with the defibrillator used on their individual institutions.

**Defibrillator basics**

*****(See *Appendix A – Defibrillator Use Presentation* which can be used as a guide as well)

- 1. Attach the pads
     1. Review of connecting pads to the defibrillator machine - this can be confusing, but is incredibly important
     2. Review of where/how to place pads on patient
  2. On/Off and Different Modes
  3. How to Defibrillate:
     1. Energy Select - Take this opportunity to review how many joules you would use for different scenarios. Direct learners to PALS cards where this is described.
     2. Charge – Review “Charge” vs. “Analyze” on machines that may have an AED mode.
     3. Shock – Review the importance of ensuring that all team members are clear prior to delivering shock, including oxygen source.
  4. How to Synchronized Cardiovert:
     1. Take this opportunity to review when you would use synchronized cardioversion vs defibrillation. If there’s a pulse, you want to sync with it or else you could convert the patient to pulseless cardiac arrest. If not, there’s nothing to sync with and nothing to lose!
     2. Turn Sync mode on
     3. Energy Select - Take this opportunity to review how many joules you would use for different scenarios. Direct learners to PALS cards where this is described.
     4. Charge – Review “Charge” vs. “Analyze” on machines that may have an AED mode.
     5. Shock – Review importance of ensuring that all team members are clear prior to delivering shock, including the oxygen source.
     6. Discuss the Sync feature needs to be pressed for each shock
  5. How to release charge without shocking patient
  6. Other buttons on the machine: this may include adjusting the volume, printing out a recording of the ECG rhythm strip, changing ECG lead display and size, and other functionality of the defibrillator used
