# Supplementary material for: Excellence in Communication and Emergency Leadership (ExCEL): Pediatric Critical Care Resource Utilization Workshop for Residents
Source: MedEdPORTAL. 2022 Aug 16;18:11268. doi: 10.15766/mep_2374-8265.11268 (PMC9378690; doi:10.15766/mep_2374-8265.11268)
Supplement: Supplementary file 1 — Defibrillator Use Presentation.pptxCode Cart Skills Station.docxTransport Bag Skills Station.docxIntroduction to Defibrillator.docxDefibrillator Use Skills Station Cases.docxDefibrillator Use Skills Session Rhythm Strips.pptxExCEL Critical Care Workshop Surveys.docx [file mep_2374-8265.11268-s001.zip › F. Defibrillator Use Skills Session Rhythm Strips.pptx]

## Slide 1
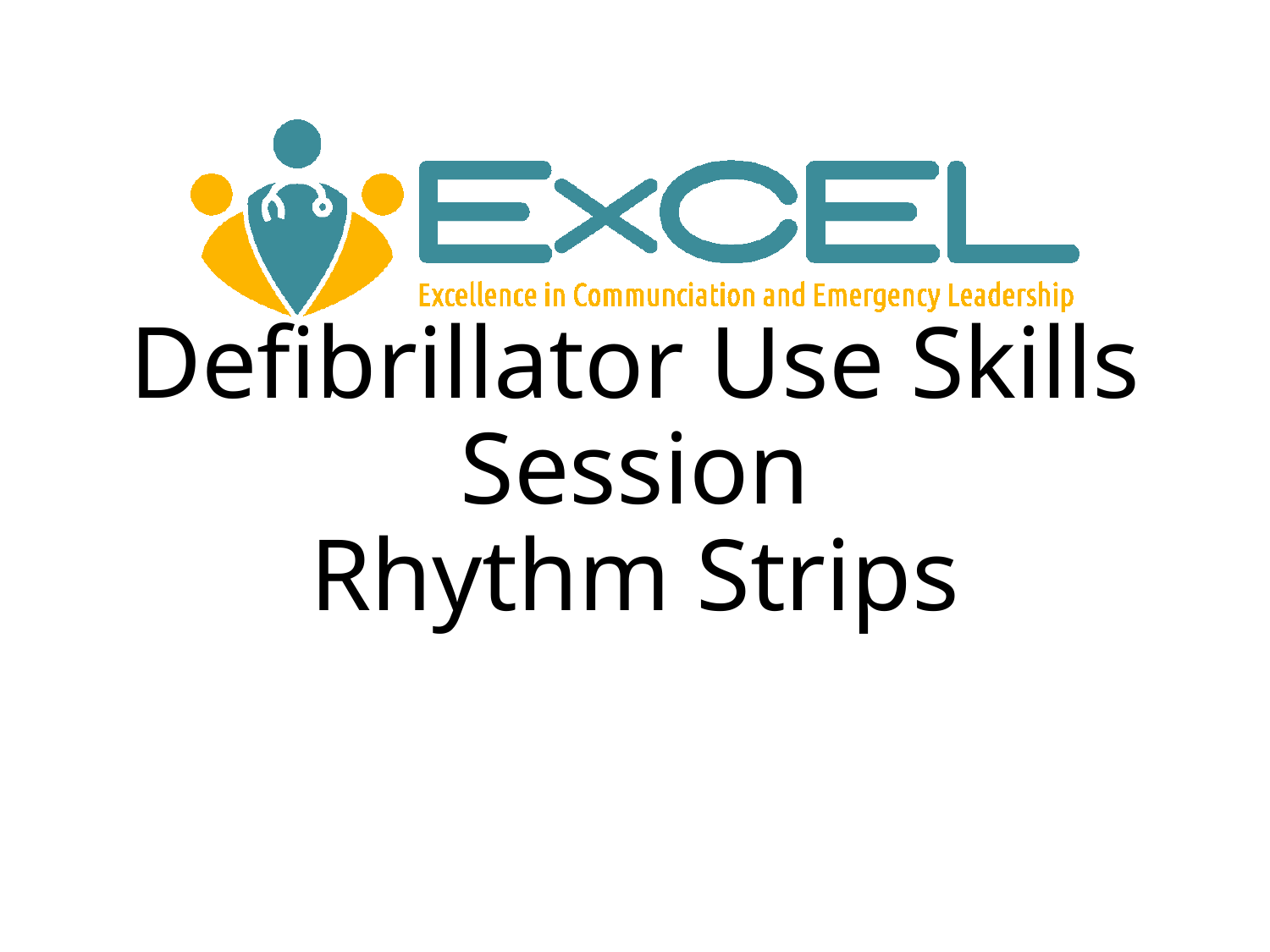

# Defibrillator Use Skills Session Rhythm Strips

## Slide 2
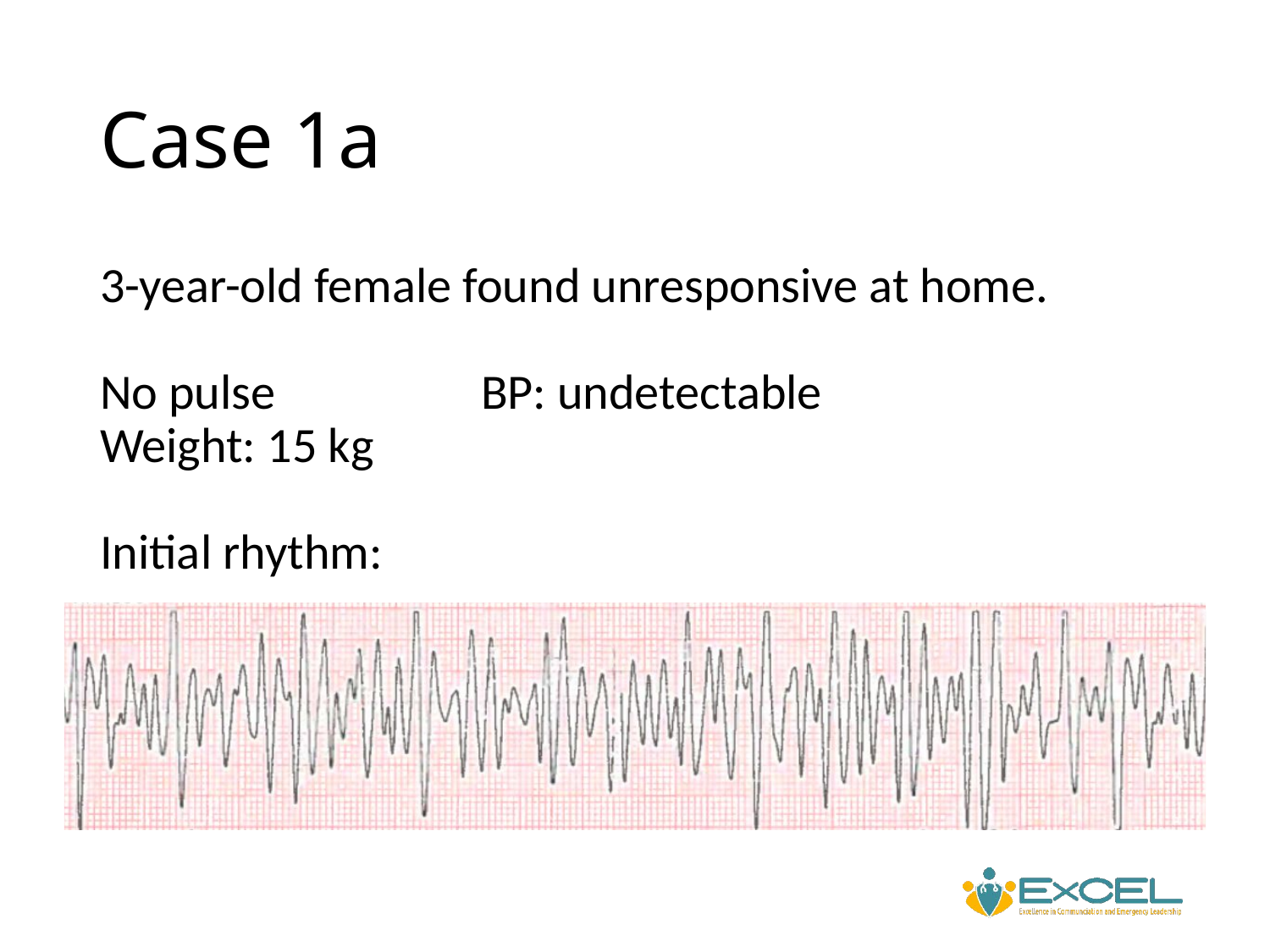

# Case 1a
3-year-old female found unresponsive at home.
No pulse 		BP: undetectable
Weight: 15 kg
Initial rhythm:

## Slide 3
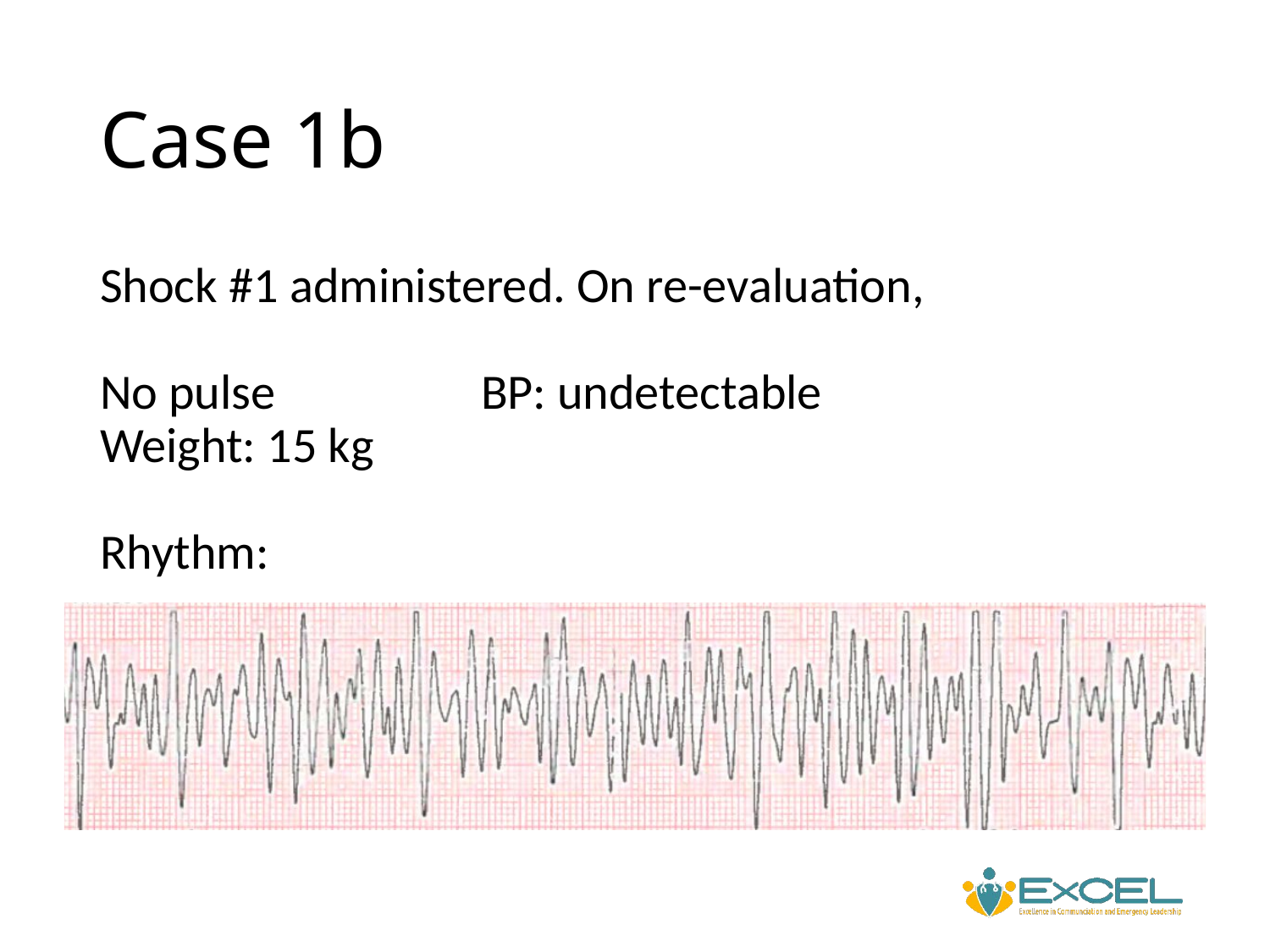

# Case 1b
Shock #1 administered. On re-evaluation,
No pulse		BP: undetectable
Weight: 15 kg
Rhythm:

## Slide 4
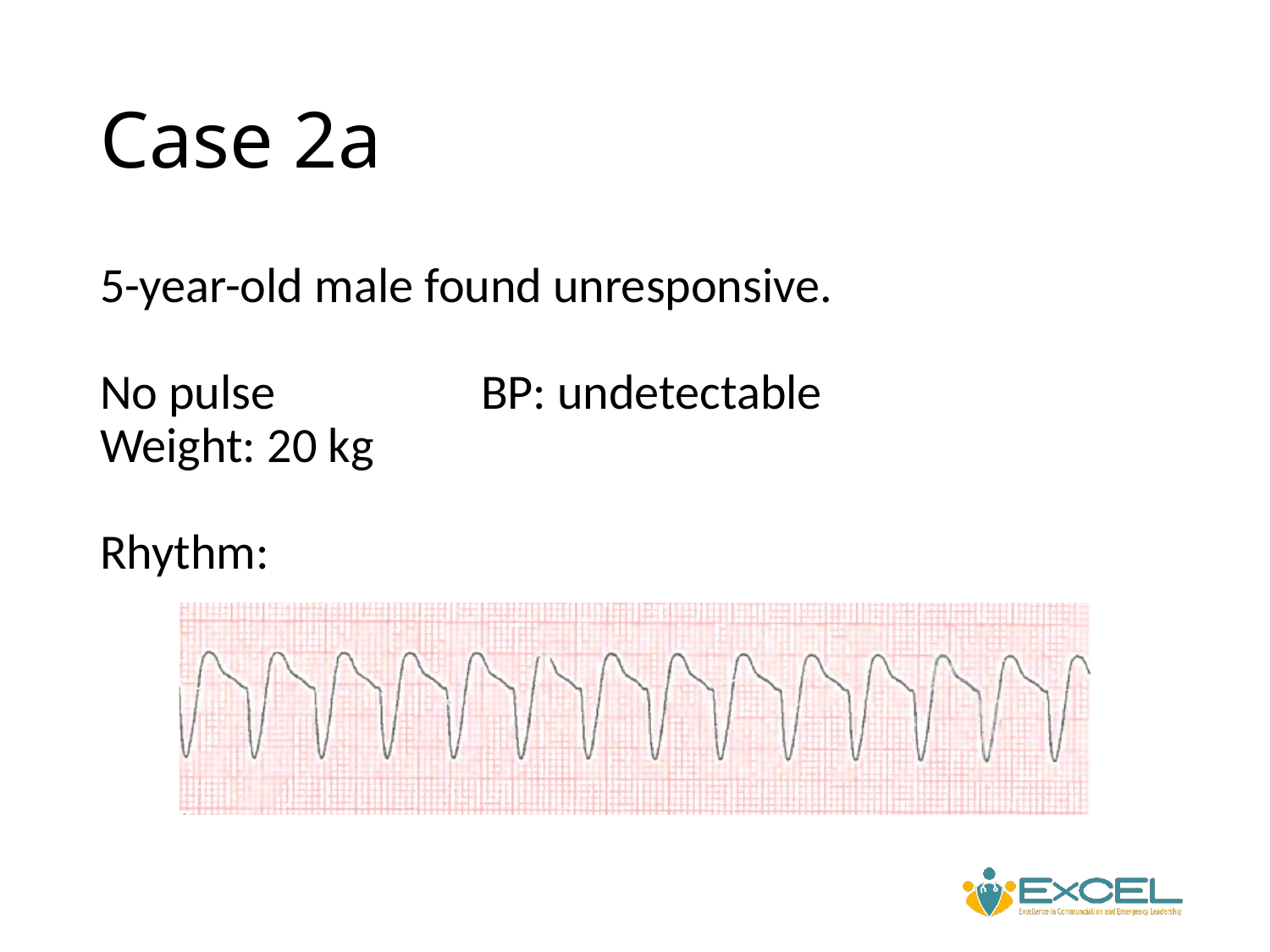

# Case 2a
5-year-old male found unresponsive.
No pulse		BP: undetectable
Weight: 20 kg
Rhythm:

## Slide 5
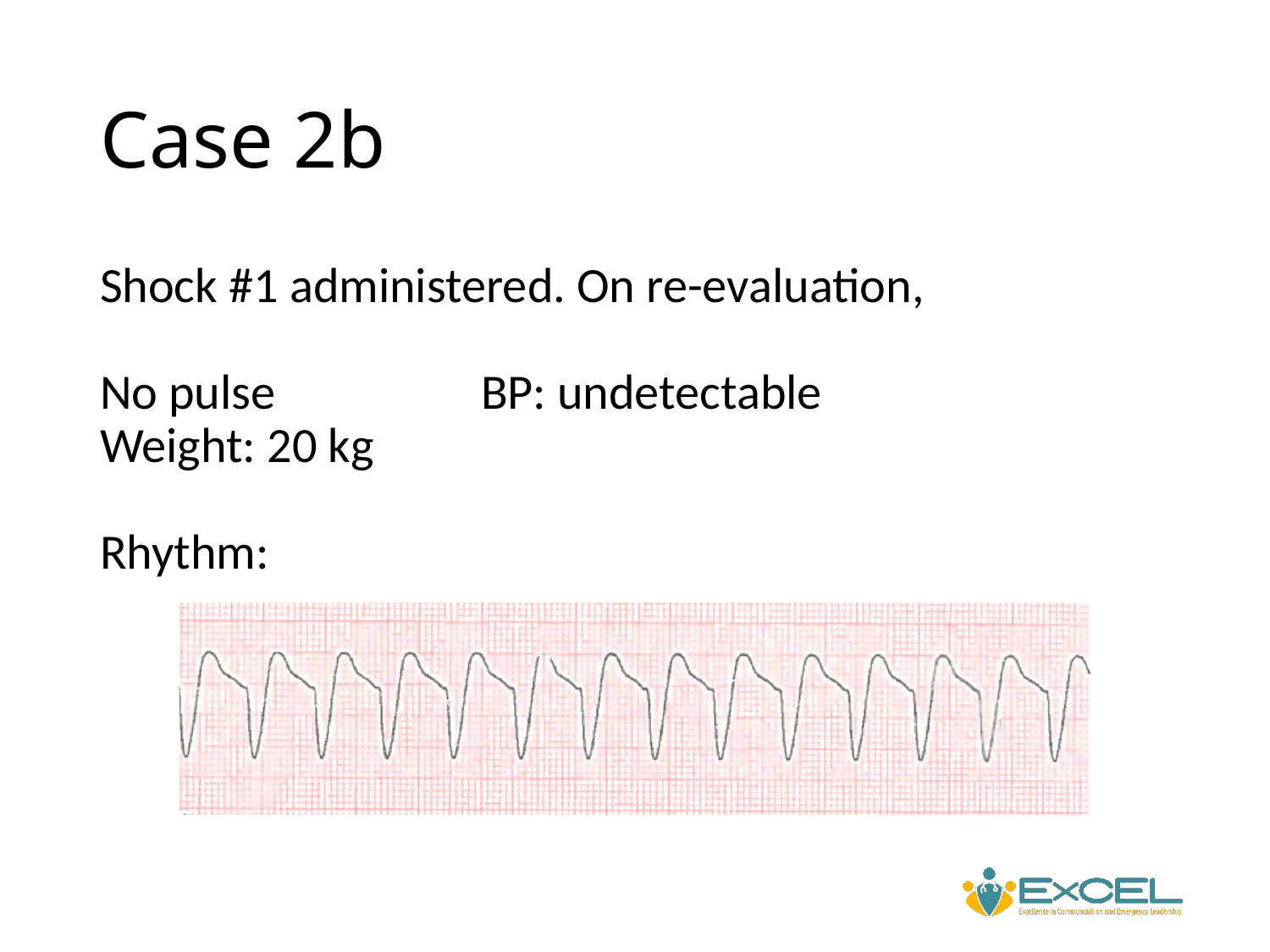

# Case 2b
Shock #1 administered. On re-evaluation,
No pulse		BP: undetectable
Weight: 20 kg
Rhythm:

## Slide 6
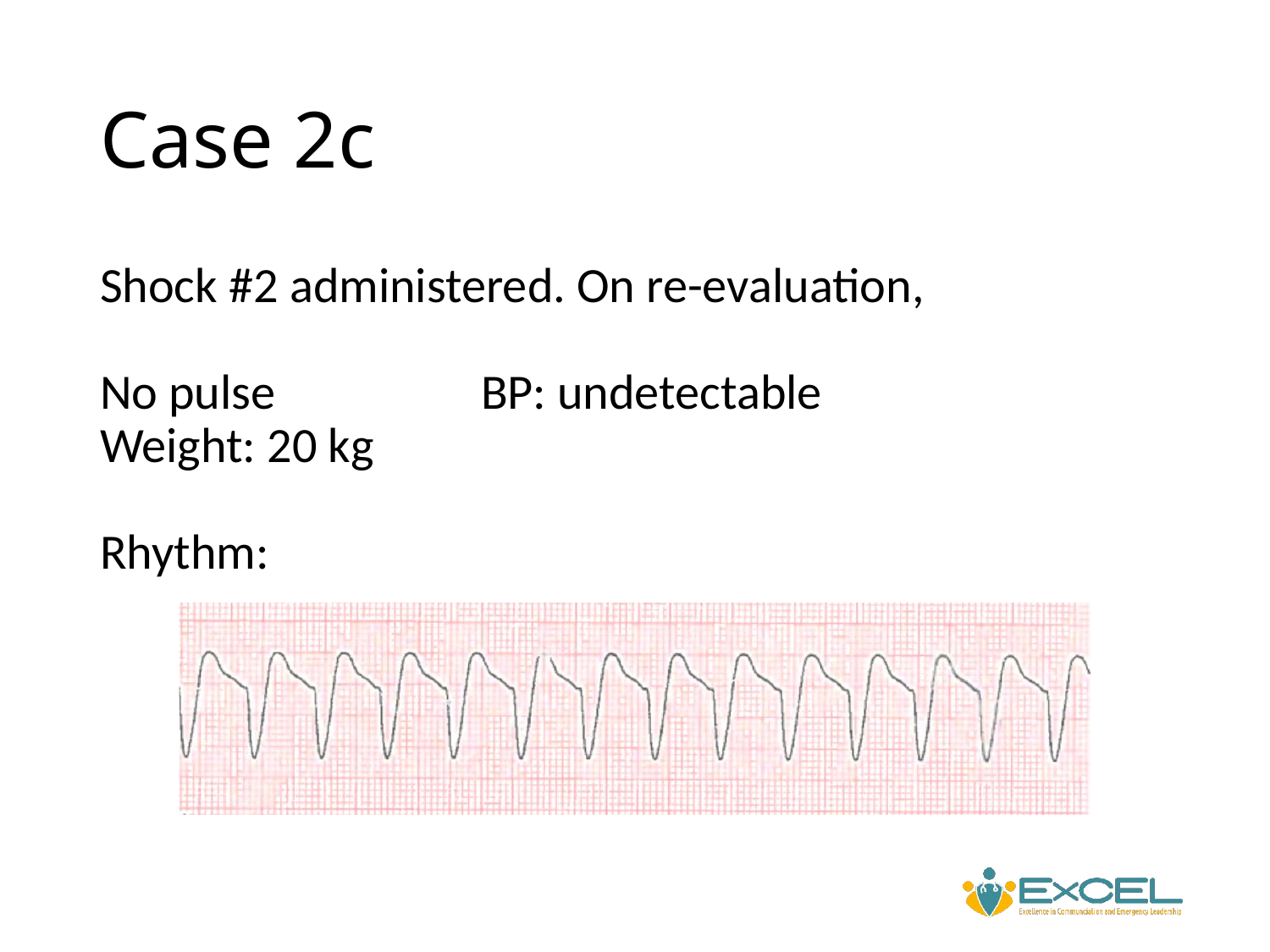

# Case 2c
Shock #2 administered. On re-evaluation,
No pulse		BP: undetectable
Weight: 20 kg
Rhythm:

## Slide 7
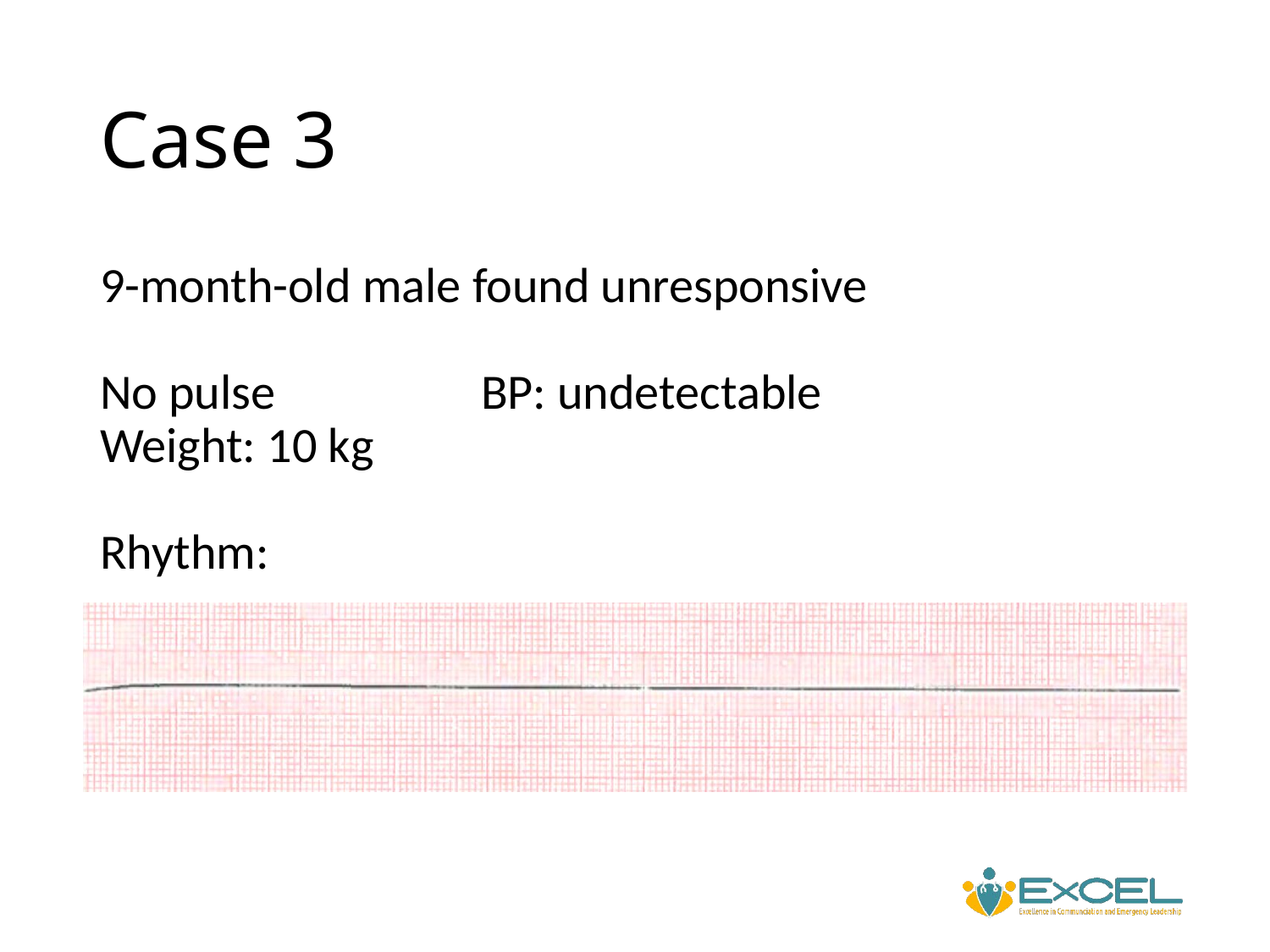

# Case 3
9-month-old male found unresponsive
No pulse 		BP: undetectable
Weight: 10 kg
Rhythm:

## Slide 8
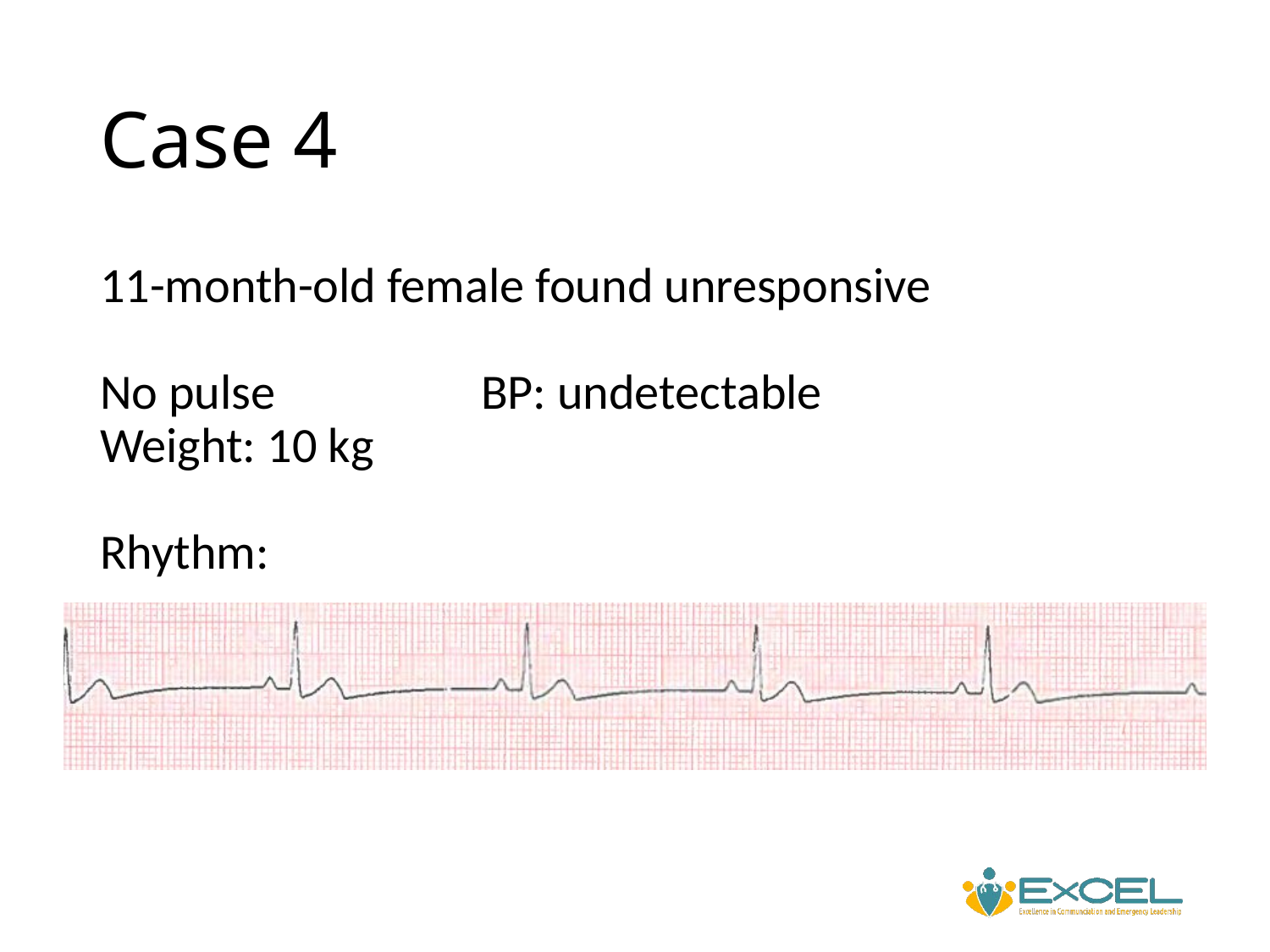

# Case 4
11-month-old female found unresponsive
No pulse 		BP: undetectable
Weight: 10 kg
Rhythm:

## Slide 9
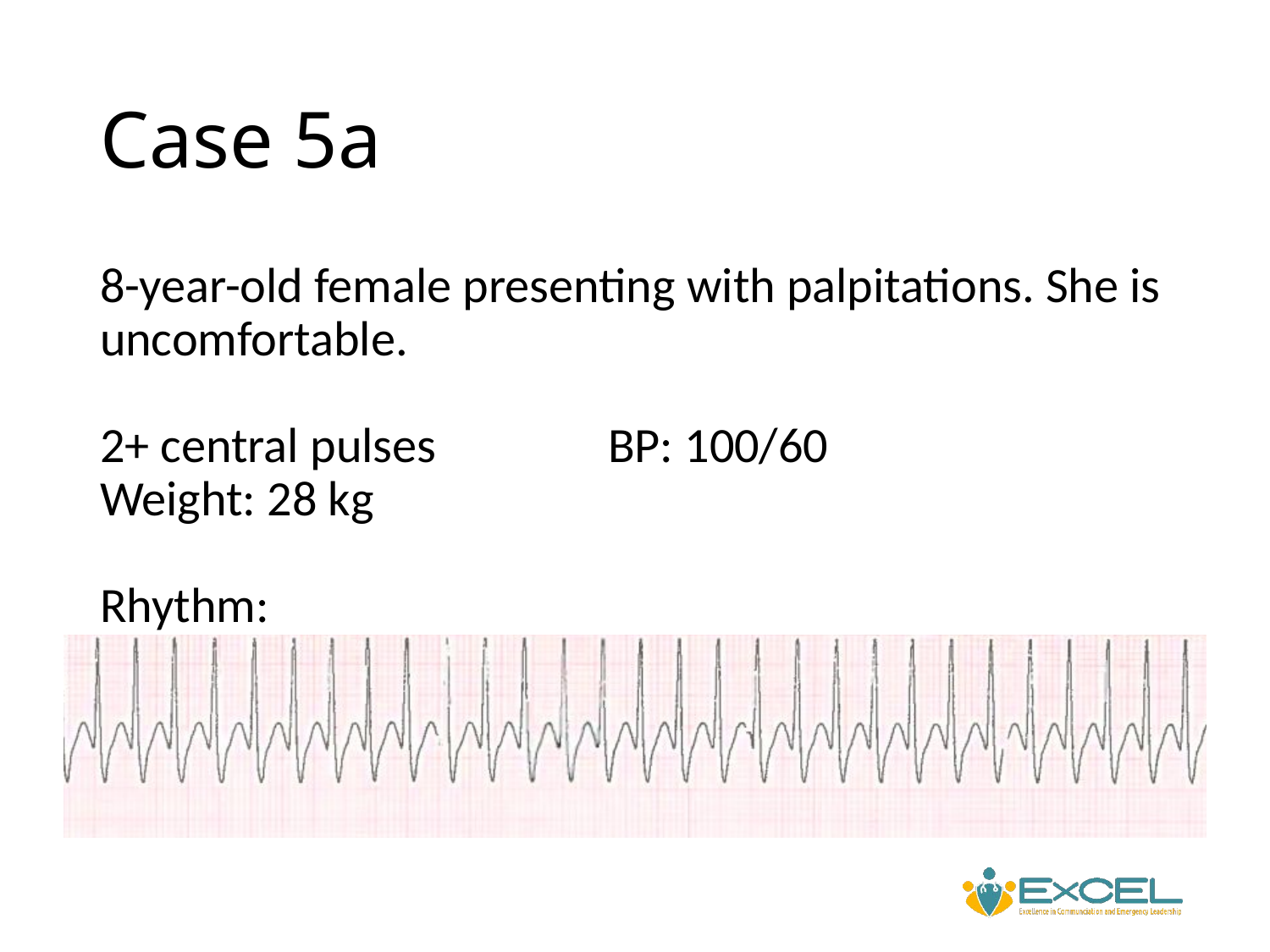

# Case 5a
8-year-old female presenting with palpitations. She is uncomfortable.
2+ central pulses 		BP: 100/60
Weight: 28 kg
Rhythm:

## Slide 10
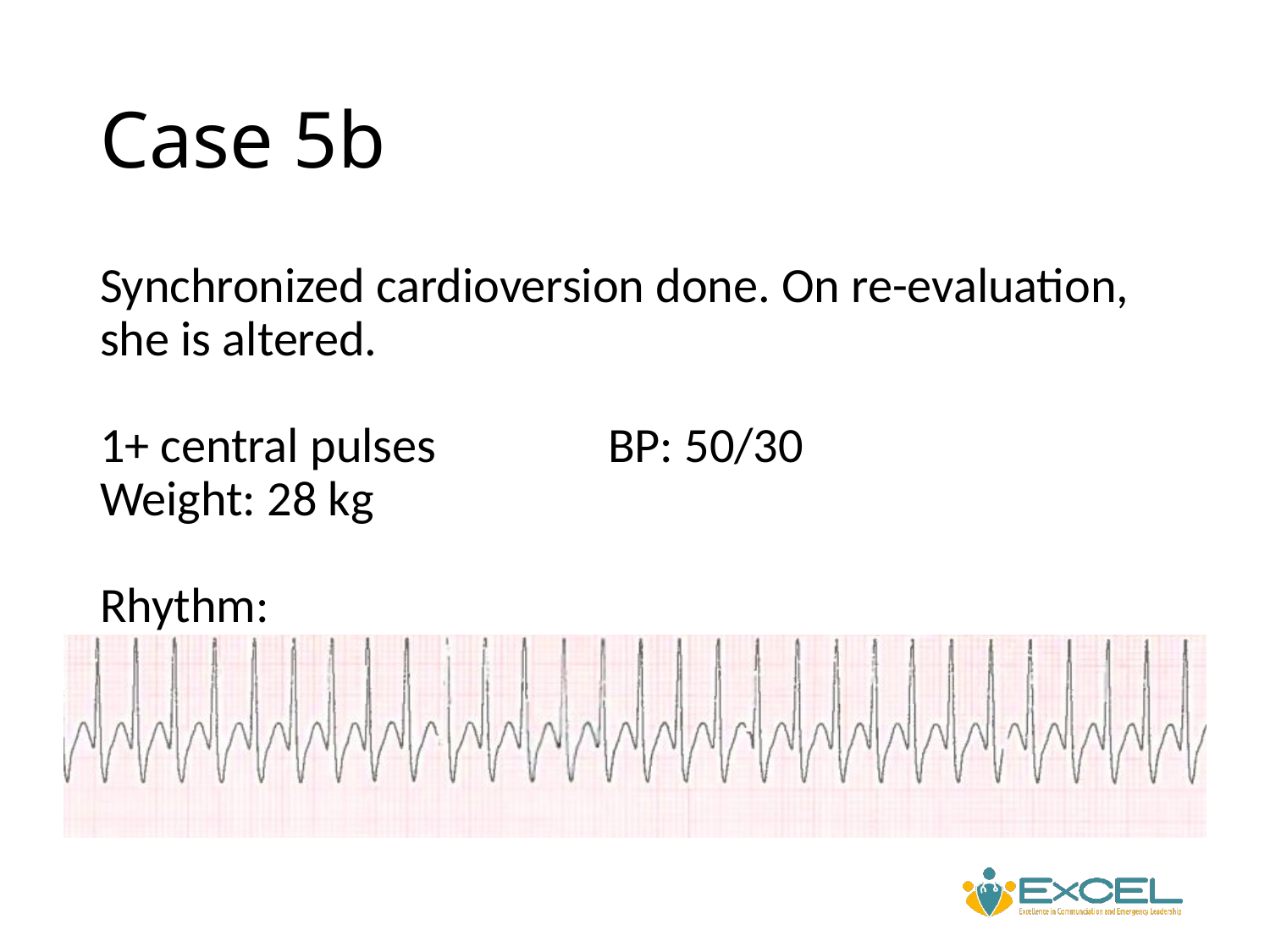

# Case 5b
Synchronized cardioversion done. On re-evaluation, she is altered.
1+ central pulses 		BP: 50/30
Weight: 28 kg
Rhythm:

## Slide 11
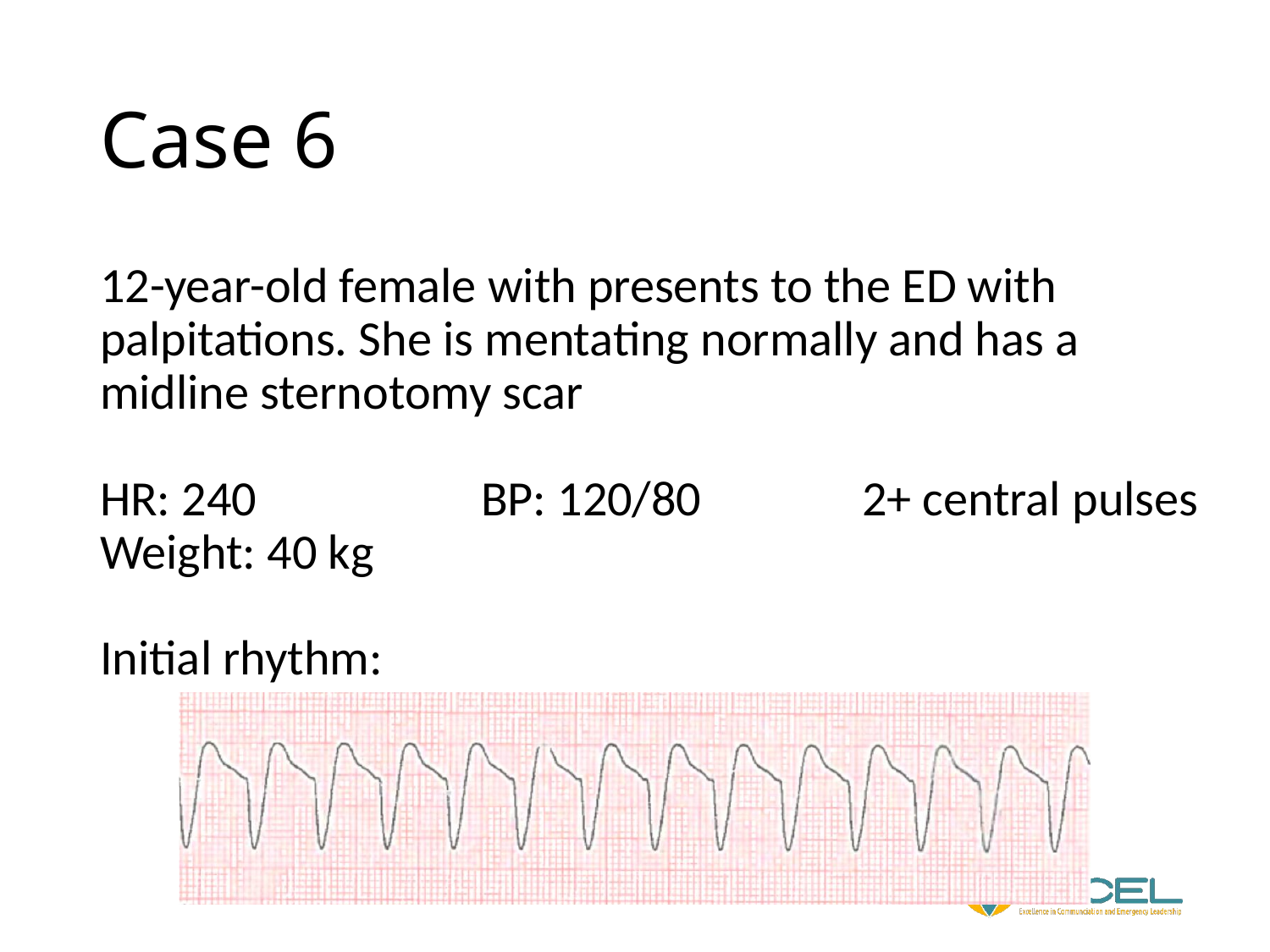

# Case 6
12-year-old female with presents to the ED with palpitations. She is mentating normally and has a midline sternotomy scar
HR: 240		BP: 120/80		2+ central pulses
Weight: 40 kg
Initial rhythm:

## Slide 12
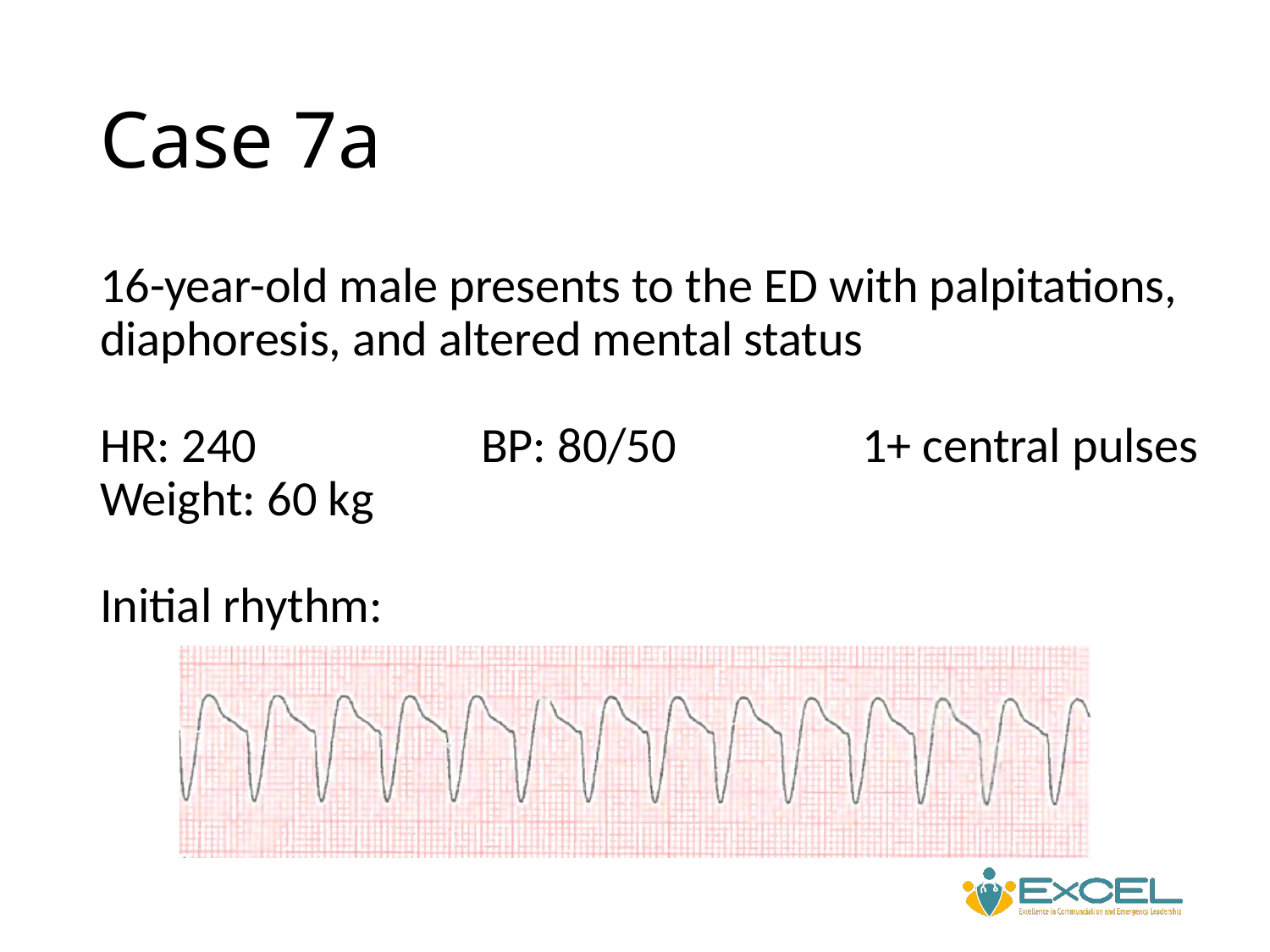

# Case 7a
16-year-old male presents to the ED with palpitations, diaphoresis, and altered mental status
HR: 240		BP: 80/50		1+ central pulses
Weight: 60 kg
Initial rhythm:

## Slide 13
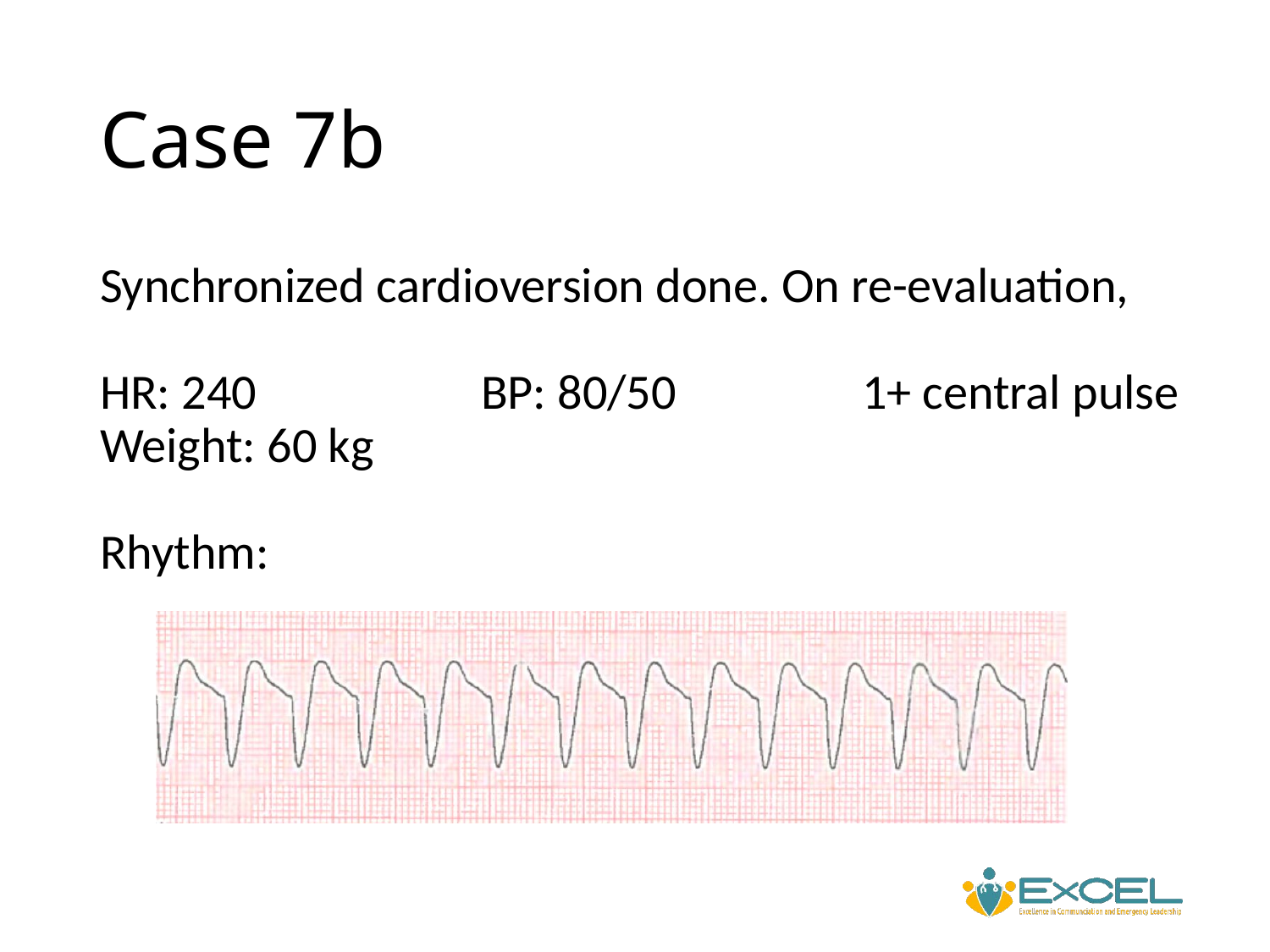

# Case 7b
Synchronized cardioversion done. On re-evaluation,
HR: 240		BP: 80/50		1+ central pulse
Weight: 60 kg
Rhythm:
