# Supplementary material for: Excellence in Communication and Emergency Leadership (ExCEL): Pediatric Critical Care Resource Utilization Workshop for Residents
Source: MedEdPORTAL. 2022 Aug 16;18:11268. doi: 10.15766/mep_2374-8265.11268 (PMC9378690; doi:10.15766/mep_2374-8265.11268)
Supplement: Supplementary file 1 — Defibrillator Use Presentation.pptxCode Cart Skills Station.docxTransport Bag Skills Station.docxIntroduction to Defibrillator.docxDefibrillator Use Skills Station Cases.docxDefibrillator Use Skills Session Rhythm Strips.pptxExCEL Critical Care Workshop Surveys.docx [file mep_2374-8265.11268-s001.zip › G. ExCEL Critical Care Workshop Surveys.docx]

**ExCEL Workshop Survey – Pediatric Code Cart Skills Station**

1. I am a:

☐ Pediatric Intern ☐ Pediatric Resident ☐ EM Intern ☐ EM Resident

☐ Family Medicine Resident ☐ Medical Student

☐ Other _______________________

1. Have you participated in this ExCEL skills session in the past?

☐ Yes ☐ No ☐ Unsure

1. When was the last time you took PALS?

☐ <6 months ☐ 6 months – less than 1 year ☐ 1-2 years ☐ Unsure

**Please rate your agreement with the following statements:**

|  |  | Strongly Disagree | Disagree | Neither Agree nor Disagree | Agree | Strongly Agree |
| --- | --- | --- | --- | --- | --- | --- |
| 4. | This skills station was relevant to my work. | □ | □ | □ | □ | □ |
| 5. | This skills station was effective in teaching me the equipment contained in the code cart. | □ | □ | □ | □ | □ |

**After participating in this session, how confident are you in your ability to:**

|  |  | Very Not confident | Not confident | Neutral | Confident | Very Confident |
| --- | --- | --- | --- | --- | --- | --- |
| 6. | Recognize equipment needed for a critically ill patient? | □ | □ | □ | □ | □ |
| 7. | Identify equipment which is kept in the code cart? | □ | □ | □ | □ | □ |
| 8. | Identify equipment which is NOT kept in the code cart? | □ | □ | □ | □ | □ |

10. What did you find most helpful about this skills session?

11. What do you think could be improved upon for this skills session in the future?

12. Other comments or suggestions:

**ExCEL Workshop Survey – Pediatric Transport Bag Skills Station**

1. I am a:

☐ Pediatric Intern ☐ Pediatric Resident ☐ EM Intern ☐ EM Resident

☐ Family Medicine Resident ☐ Medical Student

☐ Other _______________________

1. Have you participated in this ExCEL skills session in the past?

☐ Yes ☐ No ☐ Unsure

1. When was the last time you took PALS?

☐ <6 months ☐ 6 months – less than 1 year ☐ 1-2 years ☐ Unsure

**Please rate your agreement with the following statements:**

|  |  | Strongly Disagree | Disagree | Neither Agree nor Disagree | Agree | Strongly Agree |
| --- | --- | --- | --- | --- | --- | --- |
| 4. | This skills station was relevant to my work. | □ | □ | □ | □ | □ |
| 5. | This skills station was effective in teaching me the equipment contained in the transport bag. | □ | □ | □ | □ | □ |

**After participating in this session, how confident are you in your ability to:**

|  |  | Very Not confident | Not confident | Neutral | Confident | Very Confident |
| --- | --- | --- | --- | --- | --- | --- |
| 6. | Recognize the equipment needed for a critically ill patient? | □ | □ | □ | □ | □ |
| 7. | Identify and locate equipment which is kept in the transport bag? | □ | □ | □ | □ | □ |
| 8. | Identify equipment which is NOT kept in the transport bag? | □ | □ | □ | □ | □ |

9. What did you find most helpful about this skills session?

10. What do you think could be improved upon for this skills session in the future?

11. Other comments or suggestions:

**ExCEL Workshop Survey – Defibrillator Use Skills Station**

1. I am a:

☐ Pediatric Intern ☐ Pediatric Resident ☐ EM Intern ☐ EM Resident

☐ Family Medicine Resident ☐ Medical Student

☐ Other _______________________

1. Have you participated in this ExCEL skills session in the past?

☐ Yes ☐ No ☐ Unsure

1. When was the last time you took PALS?

☐ <6 months ☐ 6 months – less than 1 year ☐ 1-2 years ☐ Unsure

**Please rate your agreement with the following statements:**

|  |  | Strongly Disagree | Disagree | Neither Agree nor Disagree | Agree | Strongly Agree |
| --- | --- | --- | --- | --- | --- | --- |
| 4. | This skills station was relevant to my work. | □ | □ | □ | □ | □ |
| 5. | This skills station was effective in teaching me the steps in the use of the defibrillator. | □ | □ | □ | □ | □ |

**After participating in this session, how confident are you in your ability to:**

|  |  | Very Not confident | Not confident | Neutral | Confident | Very Confident |
| --- | --- | --- | --- | --- | --- | --- |
| 6. | After participating in this skills station, how confident are you in your ability to identify Pediatric Life Support rhythms? | □ | □ | □ | □ | □ |
| 7. | After participating in this skills station, how confident are you in your ability to differentiate between shockable and non-shockable rhythms? | □ | □ | □ | □ | □ |
| 8. | Appropriately use the defibrillator for defibrillation and synchronized cardioversion? | □ | □ | □ | □ | □ |

9. What did you find most helpful about this skills session?

10. What do you think could be improved upon for this skills session in the future?

11. Other comments or suggestions:
