# Supplementary material for: Excellence in Communication and Emergency Leadership (ExCEL): Pediatric Critical Care Resource Utilization Workshop for Residents
Source: MedEdPORTAL. 2022 Aug 16;18:11268. doi: 10.15766/mep_2374-8265.11268 (PMC9378690; doi:10.15766/mep_2374-8265.11268)
Supplement: Supplementary file 1 — Defibrillator Use Presentation.pptxCode Cart Skills Station.docxTransport Bag Skills Station.docxIntroduction to Defibrillator.docxDefibrillator Use Skills Station Cases.docxDefibrillator Use Skills Session Rhythm Strips.pptxExCEL Critical Care Workshop Surveys.docx [file mep_2374-8265.11268-s001.zip › E. Defibrillator Use Skills Station Cases.docx]

**Defibrillator Use Skills Session Cases**

The following curriculum is meant for use by the instructors to provide a standardized structure for review and practice of defibrillation and cardioversion.

**Scenario review:** Call resident participants up individually and each resident gets a different scenario for which to operate the defibrillator. Each scenario consists of the presence/absence of a pulse, a rhythm, the weight of the child, and any other information that might be relevant. The first several scenarios are very similar, but help to reinforce the basics. Remember, each scenario is for a *new* learner to ensure that all learners have the opportunity to get hands- on practice with the defibrillator.

Rhythms can be generated on the defibrillator through use of a rhythm generator device (such as the Nasco Life/Form® Rhythm generator). If no rhythm generator is available, rhythms can be displayed using the Rhythm Strips presentation (Appendix F).

Instructors should encourage the participants to verbally identify the rhythms, though initially they may need guidance in naming the dysrhythmia. After several repetitions of defibrillator basics, show the group a rhythm strip once to allow participants the opportunity for independent rhythm identification. Please encourage the group to assist the person operating the defibrillator with identifying the rhythm, energy dose to which to charge the defibrillator, and whether to sync, etc.

|  | **Pulse?** | **Rhythm on Monitor** | **Patient Weight** | **BP / Mental Status** | **Correct Action** |
| --- | --- | --- | --- | --- | --- |
| 1a | No | Ventricular Fibrillation | 15 kg | None | Defibrillate with 2J/kg (30 J) |
| 1b | No | Ventricular Fibrillation (after first defibrillation attempt unsuccessful) | 15 kg | None | Defibrillate with 4J/kg (60 J) |
| 2a | No | Ventricular Tachycardia | 20 kg | None | Defibrillate with 2J/kg (40 J) |
| 2b | No | Ventricular Tachycardia (after first defibrillation attempt unsuccessful) | 20 kg | None | Defibrillate with 4J/kg (80 J) |
| 2c | No | Ventricular Tachycardia (after second defibrillation attempt unsuccessful) | 20 kg | None | Defibrillate with 6J/kg (120 J) *Ask participant what max J they would deliver could be (10J/kg or adult dose) |
| 3 | No | Asystole | 10 kg | None | No shock |
| 4 | No | Sinus tachycardia (PEA) | 10 kg | None | No shock |
| 5a | Yes | Supraventricular Tachycardia (SVT) | 28 kg | 100/60, Uncomfortable | Vagal Maneuvers 🡪 Adenosine 🡪 Synchronized Cardioversion 0.5-1 J/kg (14-28 J) |
| 5b | Yes | Supraventricular Tachycardia (after first attempted sync cardioversion unsuccessful) | 28 kg | 50/30, Altered | Synchronized Cardioversion 2 J/kg (56 J) |
| 6 | Yes | Ventricular Tachycardia | 40 kg | 120/80, Normal | No shock; Consider adenosine/amiodarone/procainamide in conjunction with cardiologist |
| 7a | Yes | Ventricular Tachycardia | 60 kg | 80/50, Altered | Synchronized Cardioversion 0.5-1 J/kg (30-60 J) |
| 7b | Yes | Ventricular Tachycardia (first attempted sync cardioversion unsuccessful) | 60 kg | 80/50, Altered | Synchronized Cardioversion 2 J/kg (120 J) |
